# Calcium Dynamics in Hypothalamic Paraventricular Oxytocin Neurons and Astrocytes Associated with Social and Stress Stimuli

**DOI:** 10.1523/ENEURO.0196-24.2025

**Published:** 2025-05-08

**Authors:** Katy Celina Sandoval, Joshua Rychlik, Katrina Y. Choe

**Affiliations:** Department of Psychology, Neuroscience and Behaviour, McMaster University, Hamilton, Ontario L8S 4K1, Canada

**Keywords:** astrocytes, fiber photometry, oxytocin, social behavior, stress

## Abstract

Activation of hypothalamic paraventricular oxytocin (OXT^PVN^) neurons by social or stress stimuli triggers OXT release to promote social investigation and buffer adverse effects of stress, respectively. Astrocytes, a type of glial cells, can bidirectionally interact with hypothalamic neurons to participate in local activity regulation within the paraventricular nucleus (PVN). It remains unknown whether contextual factors related to stimuli, as well as biological factors such as sex, influence OXT^PVN^ neuronal or astrocyte activity and/or their interactions. To address this question, we performed dual-color fiber photometry in freely behaving male and female mice to simultaneously record Ca^2+^ dynamics in OXT^PVN^ neurons and astrocytes during acute social (i.e., interactions with familiar vs. unfamiliar conspecifics) and stress (i.e., looming shadow) stimuli. During social stimuli, we observed the most pronounced Ca^2+^ changes in OXT^PVN^ neurons in females, revealing sex and familiarity context specificity. No astrocyte Ca^2+^ changes were detected in either sex regardless of conspecific familiarity. In contrast, looming shadow stress increased Ca^2+^ in both OXT^PVN^ neurons and astrocytes in both sexes during an active escape (“run”) strategy. Ca^2+^ level changes in OXT^PVN^ neurons and astrocytes were significantly correlated during social investigations in both sexes regardless of conspecific familiarity. During looming shadow, this functional coupling was only observed in females during active escape. Together, our results suggest that sex, context, and behavioral strategy serve as major factors that shape the activity of OXT^PVN^ neurons and astrocytes, as well as their functional coupling, to potentially aid the adaptive response to social or stress stimuli.

## Significance Statement

Social and stress stimuli activate paraventricular OXT (OXT^PVN^) neurons, but it remains unclear whether these responses—and those of neighboring astrocytes—are sex- or context-dependent. To investigate this, we performed dual-color fiber photometry recordings in freely behaving mice. Increased activity of OXT^PVN^ neurons, but not astrocytes, was pronounced in female mice while interacting with unfamiliar female mice. In contrast, both cell types were robustly activated while male and female mice actively escaped the looming shadow stress stimulus. OXT^PVN^-astrocyte functional coupling was more robust during social investigation compared with looming shadow stress. Our findings suggest that sex, context, and behavioral strategy shape OXT^PVN^ neuron and astrocyte activity, as well as their coupling, to support adaptive responses to social or stress stimuli.

## Introduction

The ability to modify behavior in response to social and stress stimuli is a key factor for survival (Chen and Hong, 2018). A major evolutionary mechanism that facilitates this process is the neuromodulatory peptide oxytocin (OXT), synthesized by neurons in the hypothalamic paraventricular and supraoptic nuclei and released both peripherally and centrally (Carter, 2014). The peripheral release of OXT from the posterior pituitary is activity-dependent and critical for physiological functions such as parturition and lactation (Ludwig and Leng, 2006; Viero et al., 2010). On the other hand, centrally released OXT plays a crucial role in promoting positive social interactions (Ferguson et al., 2001; Viero et al., 2010; Oettl et al., 2016). Specifically, animals exhibit increased paraventricular OXT (OXT^PVN^) neuronal activity at the single cellular (Resendez et al., 2020) and population levels (Zhan et al., 2024) while socially investigating another individual of the same species (i.e., conspecific). In particular, one aspect of social investigation specifically implicating OXT is social recognition, which is an ability to differentiate between familiar and unfamiliar conspecifics (Ferguson et al., 2001; Gabor et al., 2012; Oettl et al., 2016). Despite this well-known role of OXT, whether conspecific familiarity produces differential effects on OXT^PVN^ neuronal activity during social investigation remains unknown.

Growing evidence has revealed yet another important role of OXT in stress buffering (Uvnäs-Moberg et al., 2024). Specifically, OXT has been shown to reduce the activity of the hypothalamic–pituitary–adrenal (HPA) axis and mitigate anxiety-like behaviors during physiological and psychological stress (Windle et al., 1997; Amico et al., 2004, 2008; Mantella et al., 2005; Zoicas et al., 2014). Multiple studies have demonstrated that noxious stimuli and immobilization stress increase peripheral OXT levels (Nishioka et al., 1998; Engelmann et al., 2001; Wotjak et al., 2001; Smith and Wang, 2014; Duque-Wilckens et al., 2018), suggesting that these stress stimuli may increase OXT neuronal spiking and trigger an activity-dependent release into the periphery (Knobloch et al., 2012). Supporting this possibility, it was recently demonstrated that restraint and tail suspension stress stimuli increase OXT^PVN^ neuronal activity (Zhan et al., 2024). However, both types of stress stimuli used in this study involve physical touch, which can independently increase the spiking activity of OXT^PVN^ neurons (Tang et al., 2020), raising the question of whether stress stimuli without physical touch can still activate OXT^PVN^ neurons.

A bidirectional functional relationship exists between OXT neurons and neighboring astrocytes (Wang and Hatton, 2009), suggesting a potential role for this glial cell type to influence stimulus-evoked changes to OXT^PVN^ neuronal activity. Both cell culture and ex vivo experiments have demonstrated that astrocytes respond to nearby increases in neuronal and synaptic activity by sensing elevated extracellular concentration of potassium ions (Strohschein et al., 2011), as well as presynaptically released glutamate (Cornell-Bell et al., 1990) and neuropeptides (Di Scala-Guenot et al., 1994; Asrican et al., 2020). In particular, OXT receptor activation has been shown to increase calcium (Ca^2+^) levels in cultured hypothalamic astrocytes (Di Scala-Guenot et al., 1994), raising a possibility that dendritically released OXT in stimulated conditions (Ludwig, 1998; Ludwig and Leng, 2006; Grinevich and Neumann, 2021) can activate neighboring astrocytes within the paraventricular nucleus (PVN) and influence the activity of hypothalamic neurons in return via mechanisms such as gliotransmitter release (Panatier et al., 2006; Chen et al., 2019). Furthermore, ex vivo (Schnell et al., 2011) and in vivo (Suthard et al., 2024) studies have demonstrated that neurons and astrocytes exhibit significantly correlated fluctuations in Ca^2+^ levels, as evidence of functional coupling between the two cell types. Nonetheless, it has never been experimentally demonstrated whether this type of functional coupling exists between OXT^PVN^ neurons and astrocytes in vivo and, whether this relationship can be modified by stimuli highly pertinent to the activity of OXT^PVN^ neurons, namely, social and stress stimuli.

We hypothesized that various social and stress contexts influence the activity of OXT^PVN^ neurons and neighboring astrocytes and modify the functional coupling between the two cell types. To test this, we performed dual-color fiber photometry recordings to monitor the Ca^2+^ levels of OXT^PVN^ neurons and neighboring astrocytes in male and female mice while they experienced social (i.e., interactions with familiar vs. unfamiliar conspecifics) and stress (i.e., looming shadow) contexts. Our data show that social investigation is associated with sex- and familiarity-specific Ca^2+^ elevations in OXT^PVN^ neurons, but not in astrocytes. In contrast, looming shadow stress increased Ca^2+^ levels in both OXT^PVN^ neurons and astrocytes. Finally, we observed significantly correlated Ca^2+^ changes between OXT^PVN^ neurons and astrocytes in both social and stress stimuli in a sex-, context-, and behavior-dependent manner.

## Materials and Methods

### Mice

All animal procedures were performed in accordance with McMaster University Animal Research Ethics Board's regulations. The transgenic mouse line Oxt^tm1.1(cre)Dolsn^/J (Oxt-Cre; Wu et al., 2012) was obtained from The Jackson Laboratory (The Jackson Laboratory stock #024234) and bred in-house. Heterozygous male and female Oxt-Cre (Oxt^cre/+^) mice were obtained from C57BL/6 [wild-type (WT)] × homozygous (Oxt^cre/cre^) or WT × heterozygous crossings and used for experiments. The genotypes were determined using PCR analyses using the following primers: 5′-ACA CCG GCC TTA TTC CAA G-3′ (mutant; 13007), 5′-TTT GCA GCT CAG AAC ACT GAC-3′ (common;19178), 5′-AGC CTG CTG GAC TGT TTT TG-3′ (wild-type; 19179; The Jackson Laboratory). Mice were housed in standard cages (28 cm × 17.5 cm × 12 cm, bedding, nesting material, and a tube as enrichment object) with sex-matched littermates (maximum of four mice per cage). They were kept on a 12 h light/dark cycle with lights on at 8 A.M. at an ambient room temperature of ∼22°C with food and water available *ad libitum*. Mice were 3–5 months old when experiments began. Juvenile (3–5 weeks old) WT mice were used as a social stimulus in each social interaction paradigm.

### Stereotaxic surgery, virus injection, and optical fiber implantation

**Figure 1.**
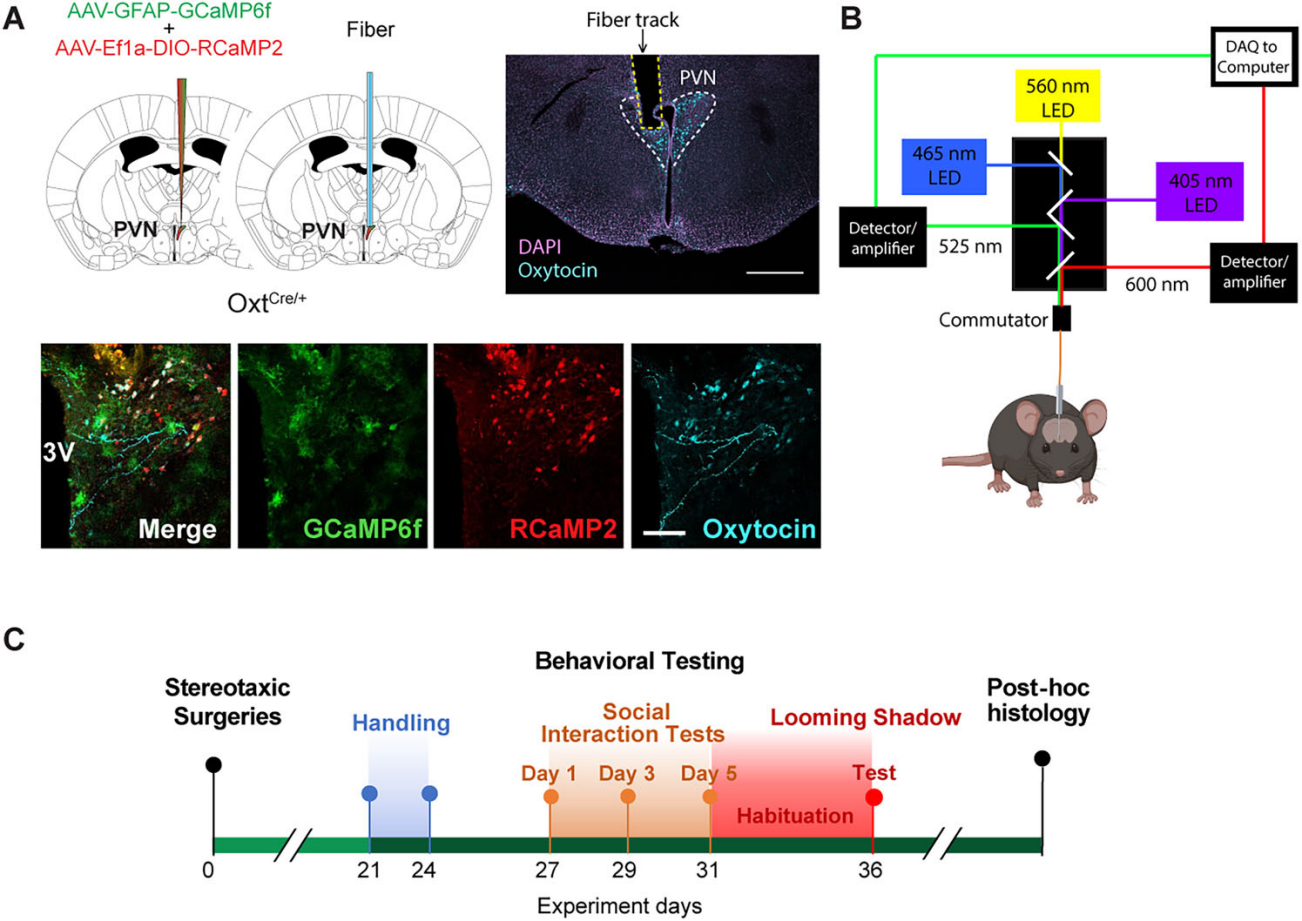
Experimental design and validation. ***A***, Top, AAV injection and fiber-optic implant strategy targeting the paraventricular nucleus (PVN) of the hypothalamus (left). Confocal micrographs of the immunostained coronal hypothalamic brain section confirm proper targeting in the PVN (right). Scale bar, 0.5 mm. Bottom, Confocal micrographs showing expression of GCaMP6f (green) in astrocyte-resembling cells and RCaMP2 (red) expression in immunostained oxytocin neurons (cyan). 3V, third ventricle. Scale bar, 100 μm. ***B***, A schematic of the dual-color fiber photometry setup. Created with BioRender.com. ***C***, Timeline of surgery, behavioral experiments, and post hoc histology. Please see Extended Data Figures 1-1 and 1-2 for additional data.

Stereotaxic surgeries were performed while mice were maintained under isoflurane anesthesia (2%) in the stereotaxic apparatus to target the PVN in the right hemisphere (anterior–posterior, −0.56 mm; lateral, +0.2 mm; dorsal–ventral, −4.8 mm). To enable dual-color fiber photometry recording of OXT neurons and neighboring astrocytes, equal volumes of adeno-associated virus (AAV)2/5-promGFAP-GCaMP6f and AAV2/9-EF1a-DIO-RCaMP2 (3.5 × 10^12^ and 2.2 × 10^13^ GC/ml, respectively, Canadian Neurophotonics Platform Viral Vector Core, Laval University) were combined into a mixture (0.6 μl total) and injected at a rate of 0.12 μl per minute into the PVN of Oxt-Cre mice using a 1 μl Neuros syringe (The Hamilton Company). The needle was slowly withdrawn after the injected virus mixture was allowed to diffuse for 5 min. Next, a 0.48 NA, 400-μm-diameter fiber-optic cannula (Doric Lenses) was implanted to target the site of virus injection using the same coordinates as above. The ferrule was attached to the skull using Metabond (Parkell) and dental cement. Mice were injected with 5 mg/kg carprofen for 3 d postsurgery. Mice were group housed postsurgery until experiments began after ∼3 weeks. For a schematic of the virus injection and fiber implant surgery, see Figure 1*A*.

10.1523/ENEURO.0196-24.2025.f1-1 Figure 1-1 Graphical representation of optic fiber implants targeting the PVN. Each color represents an individual mouse. Supports Fig .1. Download Figure 1-1, TIF file.

**Figure 2.**
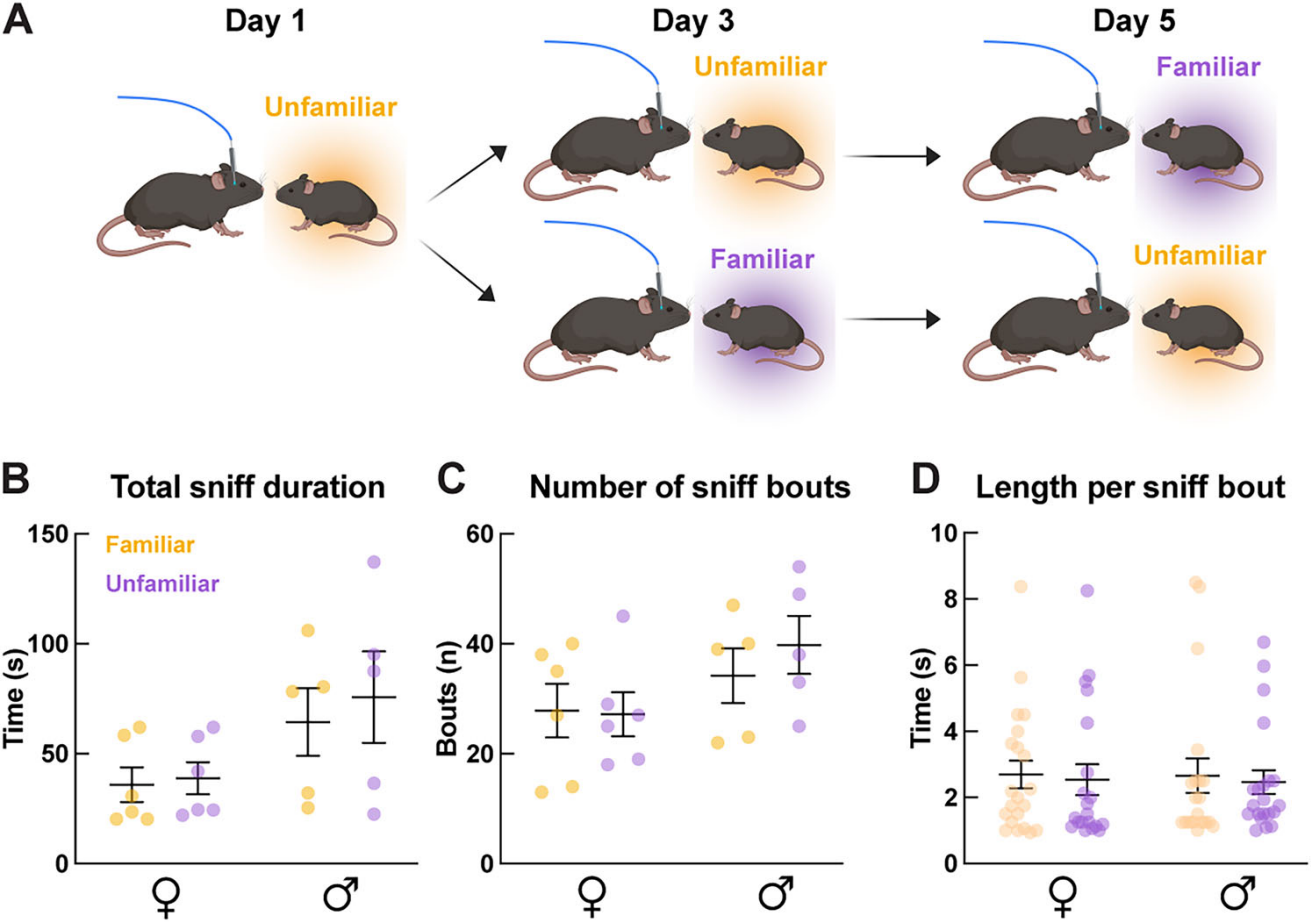
Home cage social interaction schematics and behavioral results. ***A***, A schematic of the home cage social interaction paradigm involving 3 separate days of interacting with familiar and unfamiliar juvenile conspecifics. Created with BioRender.com. ***B***–***D***, Comparisons of social sniff characteristics (i.e., total sniff duration, number of sniff bouts, and length per sniff bout) between male versus female and familiar versus unfamiliar conspecifics. No significant differences were observed.

10.1523/ENEURO.0196-24.2025.f1-2 Figure 1-2 Histological confirmation of co-expression specificity for GCaMP6 and RCaMP2. Confocal images of immunostained PVN sections displaying specificity of GCaMP6 expression in GFAP + astrocytes (A-C) and RCaMP2 in OXT-expressing cells (**D-F**). **G**, Plot shows a high (∼90%) level of coexpression percentage only for cells expressing both GCaMP6 and GFAP and those expressing RCaMP2 and OXT, but virtually none expressing other combinations. Scale bar = 100 μm. Supports Fig .1. Download Figure 1-2, TIF file.

### Dual-color fiber photometry and video recording

Fiber photometry was used to record Ca^2+^ transients from PVN astrocytes and OXT neurons in freely moving mice. A Doric fiber photometry system (see Fig. 1*B* for schematic) with three light-emitting diodes (LEDs; isosbestic, 405 nm; GCaMP6, 470 nm; RCaMP2, 560 nm) and a six-port fluorescence minicube (in nm; isosbestic excitation filters, 400–410; GCaMP6 excitation filter, 460–490; GCaMP emission filter, 500–540; RCaMP2 excitation filter, 555–570; RCaMP2 emission filter, 580–680) was used to perform the recordings. Fluorescence signals were retrieved at a 1 kHz sampling rate. Prior to behavioral assays, the intensity of each LED was modulated to be between 10–15 μW (isosbestic) and 30–50 μW (GCaMP6f and RCaMP2) as this range allows for excitation of genetically encoded calcium indicator (GECI) with limited photobleaching (Zhan et al., 2024). The frequencies of LED light sources were modulated at the following frequencies (in Hz): isosbestic, 208.616; GCaMP6, 333.786; and RCaMP2, 572.205. Optic cannulae-implanted experimental mice were coupled to a low autofluorescence patch cord connected to the fiber photometry system through a rotary joint (Doric Lenses), enabling free movement. The excitation light was directed to the animal using a mono fiber-optic patch cord. An overhead camera (Chameleon3 CM3-U3-31S4M; Sony IMX265, 1/1.8” Mono CMOS sensor) fixed to the ceiling above the apparatus was used to video record home cage social interactions. The same camera was placed on the side of the apparatus to video record the looming shadow task. Video recordings were time-locked to fiber photometry recordings at a rate of 16 frames/s.

### Behavioral assays

Mice were handled for 3 consecutive days preceding behavior assays, for 3–5 min on each day, to allow for mouse-to-experimenter habituation. Prior to conducting behavioral assays, mice were individually placed in a holding cage with some pellets, and water squirted on the pellets inside the cage. Mice were subjected to a 1 h habituation period in the dark to ease the transition to the behavioral experimental settings with dim (∼2 lux, social interaction assay) or no room light (looming shadow task). During both habituation and behavioral testing periods, a white noise machine at ∼70 dB and an air filter (Honeywell) were operational to reduce any extraneous noise and prevent accumulation of odors and mouse dander. All tests were conducted during the light phase of the light/dark cycle between 11 A.M. and 4 P.M. For a diagram of the experimental timeline, see Figure 1*C*.

#### Home cage social interaction assay

To assess responses to social stimuli, mice were placed in a neutral home cage setting where they were free to roam and interact with a social stimulus mouse. One week prior to recording, a same-sex juvenile WT mouse (3–5 weeks old) was placed in the home cage of the experimental mouse to serve as a familiar stimulus mouse. Another same-sex juvenile WT mouse (3–5 weeks old) from a different cage was selected to be used as an unfamiliar stimulus mouse. This juvenile age group was chosen for the stimulus mice to minimize aggressive interactions, which can be pronounced in interactions between adult males. For all social interaction trials, mice were placed in one of three home cage bottoms (13 cm × 12 cm × 10 cm), placed side-by-side, and separated by opaque walls to reduce mouse visibility between the cages. New home cage bottoms with clean bedding were used for each experiment. The experimental mouse was placed in the left home cage, and the stimulus mouse (familiar or unfamiliar) was placed in the right home cage for 10 min habituation. Then, both mice were placed in the middle home cage at the same time and allowed to freely interact for 10 min. The experiments spanned over 3 separate days, with a 1 d break in between experimental days. On day 1, experimental mice interacted with unfamiliar stimulus mice to allow habituation to the experimental setting. On day 2, no behavioral assay was performed. On day 3, experimental mice were randomly placed into two groups to interact with either a familiar or unfamiliar stimulus mouse. On day 4, no behavioral assay was performed. On day 5, the stimulus mouse groups (familiar vs. unfamiliar stimulus mice) were switched to counterbalance.

#### The looming shadow stress task

To assess stress response, the looming shadow task was performed as previously described (Daviu et al., 2020), with some modifications as described below. Experimental mice were individually placed in an empty home cage [13 cm (l) × 12 cm (w) × 10 cm (h)] with a 21-inch LCD monitor placed on top of the home cage and facing downward to provide visual stimuli in the form of a looming shadow. A small shelter [8 cm (l) × 6 cm (w) × 8 cm (h)] was placed in the home cage. Experimental mice were habituated to the apparatus over 5 consecutive days for 10 min to familiarize the mouse with the light from the LCD monitor and the location of the shelter. On experiment day, experimental mice were habituated in the apparatus for 10 min before the experimenter triggered the appearance of the looming shadow. Each visual stimulus was presented at ≥1 min intervals where it started with a 2 cm black disc for 3 s, which then expanded to a total diameter of 20 cm over 2 s, and finally remained for 3 s. A total of 6–8 stimulus presentations were triggered in the span of 10 min.

### Post hoc histology

After completing the behavioral experiments, experimental mice were transcardially perfused with 1× phosphate-buffered saline (PBS) and then with 4% paraformaldehyde solution (PFA). The brains were extracted, postfixed in 4% PFA overnight, and then washed and stored in 1× PBS with 0.01% sodium azide. Brains were then sectioned (50 μm) on a vibratome and imaged on an inverted confocal microscope (Nikon A1R; Centre for Advanced Light Microscopy, McMaster University) with a 10× or 20× objective to confirm correct fiber targeting and to validate the expression of GCaMP6 in astrocytes and RCaMP2 in OXT neurons. A subset of sections were stained with primary antibodies: goat polyclonal anti-GFP (1:1,000, EnCor Biotechnology) and rabbit polyclonal anti-RFP (1:1,000, Rockland Immunochemicals) to identify GCaMP6f and RCaMP2, respectively, together with either mouse monoclonal anti-OXT neurophysin (1:1,000; Millipore) or mouse monoclonal GFAP (1:500, BioLegend) for confirmation of cell types. The following secondary antibodies were used: donkey anti-goat Alexa 488, donkey anti-rabbit Alexa 555, and donkey anti-mouse Alexa 647 secondary antibodies (all at 1:500; Thermo Fisher Scientific). Mice were excluded from further analysis if the implant target was determined to be outside of the PVN. Extended Data Figure 1-1 illustrates the post hoc assessment of implant targeting for mice included in the final dataset. Extended Data Figure 1-2 shows the cell-type–specific expression of the two GECIs.

### Analysis

#### Behavioral video analysis

Video recordings were manually analyzed using BORIS software (Friard and Gamba, 2016) by a blinded experimenter. For analysis of social interactions, sniffing behaviors of the experimental mouse toward the stimulus mouse were scored. For analysis of the looming shadow task, behavioral responses were classified into three categories: run (flight to shelter), freeze (no movement), or no response (neither ran nor froze). For social interaction analysis, only sniff events lasting ≥1 s were considered for further analysis. Additionally, mice with a total event count of ≤3 were excluded from the analysis. This resulted in the exclusion of three mice from further analysis. For looming shadow analysis, behavioral responses occurring within the 8 s duration of the shadow stimulus were determined as stimulus-elicited responses.

#### Fiber photometry data analysis

Recording data were exported to Matlab R2021b (MathWorks) for offline data analysis using the Fiber Photometry Analysis (FPA) tool (Molina, 2019). Each signal was individually fit with a polynomial curve for baseline correction. Motion artifacts were corrected by subtracting the isosbestic signal (triggered by 405 nm LED) from the GECI (GCaMP6f and RCaMP2, triggered by 465 nm and 560 nm LEDs, respectively) signals at each time point. A low-pass filter of 0.3 Hz was applied to each signal. The *z*-score was calculated by the following equation: $z=(F-F_{0})/\sigma F$, where *F* is the test signal and $F_{0}$ and σF are the mean and the standard deviation of the basal signal, respectively. The start times (*t* = 0 s) of the individual events (i.e., social sniff initiation or appearance of a 2 cm black disc corresponding to the start of looming shadow sequence, quantified in BORIS as described above) were synchronized to the fluorescence signal expressed as *z*-scores. The *z*-scores for individual events were extracted from *t* = −8 to 5 s (social sniff) or *t* = −5 to 15 s (looming shadow stimulus) for further analysis. For data representation and correlation analyses (below), the *z*-score for each trial was uploaded to Excel where the mean signal during the baseline period (−8 to −3 s for social interactions due to the sniff stimulus being experimental mouse-initiated; −5 to 0 s for looming shadow) was subtracted from the entire time series. Two specific criteria were enacted to remove any irregular data points: baseline irregularity beyond ±0.2 *z*-score deviations from the baseline average and social epochs that were <1 s long. As the number of social interactions varied between mice, up to five sniff interactions were used for the analysis of OXT^PVN^ neuron and astrocyte responses.

#### Area under the curve and peak analysis

Area under the curve (AUC) analyses were performed using GraphPad Prism 10 software. For each trace with social interaction, the curve from each trial that crossed *t* = 0 s was selected. Peak *z*-score and time of peak values were extracted for the chosen curve. Peaks that were <5% of the distance from minimum to maximum *Y* were ignored. For looming shadow, the curve from each trial that included the 5–8 s period was selected. Peaks that were <10% of the distance from minimum to maximum *Y* were ignored. The time of peak was calculated based on the curve identified by the AUC analysis. For looming shadow recordings, *z*-scores between *t* = 5–8 s and *t* = 8.01–15 s were averaged to calculate the peak values. These time intervals correspond to when the shadow is in full expansion and poststimulus, respectively.

#### RCaMP2–GCaMP6f correlation analysis

Peak changes to the *z*-score from each GECI were plotted to evaluate correlated responses to stimuli. Δ*z*-scores from OXT^PVN^ neurons (RCaMP2) were plotted on the *x*-axis, and those from astrocytes (GCaMP6f) were plotted on the *y*-axis. For social interaction trials, RCaMP2 peak Δ*z*-scores were extracted from the above AUC analyses, and then GCaMP6f Δ*z*-score was extracted for the same time point (i.e., time of peak defined by RCaMP2 signal). For looming shadow trials, Δ*z*-scores from each GECI were extracted by averaging the signals from *t* = 5 to 8 s and subtracting the baseline average from −5 to 0 s.

#### Histology analysis

Coexpression of cell-type markers (i.e., GFAP or OXT) with each GECI type (GCaMP6f or RCaMP2) was quantified in the following manner: 2–3 histological sections were analyzed per mouse and the cell counts from each section were combined for each mouse. The numbers of cells expressing each marker within the PVN area were quantified using the FIJI cell counter plugin (Schindelin et al., 2012). Mice expressing coexpression percentages were calculated as follows: GCaMP6+ GFAP+ coexpressing cells / GCaMP6+ cells, RCaMP2+ GFAP+ coexpressing cells / RCaMP2+ cells, GCaMP6+ OXT+ coexpressing cells / GCaMP6+ cells, RCaMP2+ OXT+ coexpressing cells / RCaMP2+ cells, GCaMP6+ RCaMP2+ coexpressing cells / GCaMP6+ cells, and GCaMP6+ RCaMP2+ coexpressing cells / RCaMP2+ cells.

#### Statistics

Traces and quantification results are represented as mean ± standard error of the mean (SEM). Statistical analyses were performed using the GraphPad Prism 10 software. The ROUT method was used to identify and eliminate outliers in social behavioral datasets (i.e., total sniff duration, number of sniff bouts, and average length of sniff). A two-way analysis of variance (ANOVA) with [sex (male vs. female) × familiarity (familiar vs. unfamiliar)] as factors was performed to analyze for differences in social behavior. If the social behavior data did not meet the criteria of normality (Shapiro–Wilk test), two-tailed paired *t* test was used to compare raw baseline *z*-scores to nonbaseline normalized peak *z*-scores for social interaction peak analysis. Mann–Whitney test was used for AUC comparisons due to the non-normal distribution of data. The time of peak was compared using the two-tailed unpaired *t* test for normally distributed samples and Mann–Whitney test for non-normally distributed samples. *F* test was performed to compare variances. To assess the statistical significance of AUC relative to zero, we performed the Wilcoxon signed rank test. Sex comparison of behavioral responses to the looming shadow was performed using the two-tailed unpaired *t* test. One-way repeated measures ANOVA was used to compare raw *z*-scores of epochs (baseline, *t* = −5 to 0 s; looming, *t* = 5–8 s; poststimulus, *t* = 8–15 s) during the looming shadow stress task, followed by the two-stage linear step-up false discovery rate (FDR) method by Benjamini, Krieger, and Yekutieli as a post hoc test. A simple linear regression test was performed on GraphPad Prism 10, where Δ*z*-scores from OXT neurons (RCaMP2) were plotted on the *x*-axis and those from astrocytes (GCaMP6f) were plotted on the *y*-axis. *p*-values or adjusted *p*-values (Q) < 0.05 were considered statistically significant.

## Results

Using dual-color in vivo fiber photometry in mice expressing two types of GECIs, we simultaneously recorded the Ca^2+^ levels of OXT^PVN^ neurons and neighboring astrocytes as a proxy of their population activity. Given that OXT modulation of behavioral responses to social (Dumais et al., 2016) and stress (Mantella et al., 2003) stimuli can be sex-dependent, we performed our investigations in both male and female mice. Expression of RCaMP2 (Kim et al., 2016; Wu et al., 2022), a red GECI, was directed to OXT neurons using a Cre-dependent strategy (AAV2/9-EF1a-DIO-RCaMP2 in Oxt-Cre mice), and expression of GCaMP6f (Chen et al., 2013; Gunaydin et al., 2014; Wu et al., 2022), a green GECI, was driven by the glial fibrillary acidic protein (GFAP) promoter to target astrocytes (AAV2/5-promGFAP-GCaMP6f; Fig. 1*A*). Correct fiber targeting and GECI expression were validated with post hoc histology combined with immunostaining (Fig. 1*A*, Extended Data Fig. 1-1). As expected, the GCaMP6f-expressing cells within the PVN had small somata and complex, branched processes, resembling protoplasmic astrocytes (Baldwin et al., 2023). RCaMP2-expressing cells had morphological features of either magnocellular neurosecretory cells, having large and round soma and a few dendrites, or parvocellular OXT neurons, with more complex dendrite branching patterns (Chen et al., 2022). Cellular identities were further confirmed by the expression of GCaMP6f in GFAP-immunoreactive cells and RCaMP2 in OXT-immunoreactive cells (Fig. 1*A*, Extended Data Fig. 1-2). Both GCaMP6f and RCaMP2 showed highly specific expression in their targeted cell types (average number of cells within the PVN and coexpression percentage, per mouse: 31.7 ± 4.4 GFAP+ GCaMP6f+ cells out of 35.3 ± 4.2 GCaMP6f+ cells, or 89.2 ± 2.9% coexpression; 0 ± 0 RCaMP2+ GFAP+ cells out of 30 ± 9.6 RCaMP2+ cells, or 0 ± 0% coexpression; 21.7 ± 1.9 RCaMP2+ OXT+ cells out of 23.0 ± 1.0 RCaMP2+ cells, or 93.8 ± 4.2% coexpression; 0 ± 0 GCaMP6f+ OXT+ cells out of 25.7 ± 5.2 GCaMP6f+ cells, or 0 ± 0% coexpression; *n* = 3 mice). Furthermore, there was minimal coexpression of GCaMP6f and RCaMP2 (1.3 ± 0.7 GCaMP6f+ RCaMP2+ cells out of 112.7 ± 10.8 total number of GCaMP6f+ or RCaMP2+ cells, or 1.3 ± 0.7% coexpression).

To enable simultaneous recording of GCaMP6f and RCaMP2 fluorescence signals, LED illumination at 465 nm (for GCaMP6f) and 560 nm (for RCaMP2) was delivered to experimental mice through a six-channel minicube connected to a unilateral fiber-optic implant via a patch cable (Fig. 1*B*). Emitted fluorescence signals from GCaMP6f and RCaMP2 were transmitted through the implanted optic fiber, separated by dichroic mirrors within the minicube, then received by dedicated detectors/amplifiers. Combining this fiber photometry setup with video recording, we simultaneously recorded Ca^2+^ levels in OXT^PVN^ neurons and astrocytes to detect changes in the Ca^2+^ level of these cell types in association with behavioral responses to social and stress stimuli (Fig. 1*C*).

### Female mice exhibit elevated Ca^2+^ in OXT^PVN^ neurons, but not astrocytes, during social investigation of unfamiliar conspecifics

Previous studies have shown that rodents prefer investigating unfamiliar conspecifics (Moy et al., 2004; Dumais et al., 2016) while others have shown the opposite (Pearson et al., 2010; Lu et al., 2019; Lu and Hu, 2021) or lack any preference (Oettl et al., 2016). To better understand the relationship between conspecific familiarity and the activity of OXT^PVN^ and astrocytes, we examined the Ca^2+^ dynamics of OXT^PVN^ neurons and astrocytes while mice engaged in free social interactions with familiar versus unfamiliar mice. Male (*n* = 5) and female (*n* = 6) mice were exposed to familiar or unfamiliar sex-matched juvenile (3–5 weeks old) conspecific in a home cage setting (Fig. 2*A*). First, we examined the social investigation behavior on its own to examine whether the familiarity of stimulus mice and sex significantly modified it. We compared the total duration, number, and average bout length of sniff interactions by experimental mice. For the total sniff duration, we did not observe a significant interaction between sex and familiarity of stimulus mice (sex × familiarity, *F*_(1,18)_ = 0.10, *p* = 0.75, two-way ANOVA) or a main effect of familiarity (*F*_(1,18)_ = 0.30, *p* = 0.59). A significant main effect attributed to sex (*F*_(1,18)_ = 6.25, *p* = 0.022) was observed, but post hoc comparisons showed no group differences [Fig. 2*B*; in s, familiar, 35.94 ± 7.87 (females), 64.49 ± 15.38 (males); *Q* = 0.39; unfamiliar, 38.85 ± 7.32 (females), 75.81 ± 20.81 (males); *Q* = 0.26; FDR-corrected]. Furthermore, no sex- or familiarity-specific differences were observed in the number of sniff bouts [Fig. 2*C*; females, 27.83 ± 4.88 (familiar), 27.17 ± 3.99 (unfamiliar); males, 34.2 ± 4.97 (familiar), 39.8 ± 5.27 (unfamiliar); *F*_(1,18)_ = 0.43, *p* = 0.52 (sex × familiarity); *F*_(1,18)_ = 3.95, *p* = 0.062 (sex); *F*_(1,18)_ = 0.27, *p* = 0.61 (familiarity); two-way ANOVA]. Next, we compared the average length of individual sniff bouts [Fig. 2*D*; females, 2.41 ± 0.32 (familiar), 1.45 ± 0.13 (unfamiliar); males, 1.75 ± 0.17 (familiar), 1.87 ± 0.18 (unfamiliar)]. We observed no differences in the average length per sniff bout between familiar and unfamiliar stimuli [*F*_(1.78)_ = 0.0023, *p* = 0.96 (sex × familiarity); *F*_(1,78)_ = 0.017, *p* = 0.90 (sex); *F*_(1.78)_ = 0.16, *p* = 0.69 (familiarity)], consistent with previous findings (Oettl et al., 2016).

**Figure 3.**
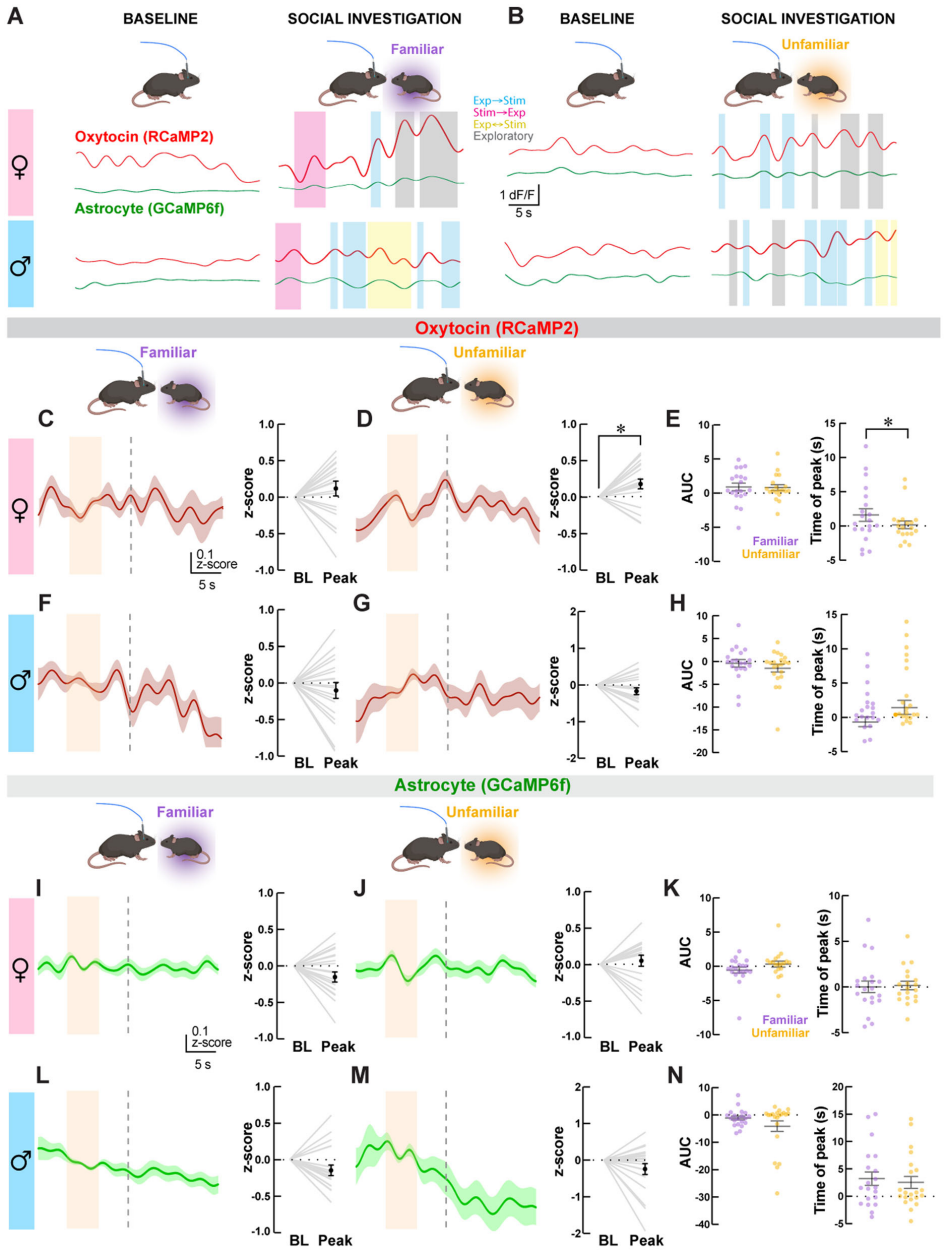
Fiber photometry recordings of paraventricular oxytocin (OXT^PVN^) neurons and astrocytes during home cage social interactions. ***A***, ***B***, Representative single traces of RCaMP2 (red) and GCaMP6f (green) recordings during social interactions. Shaded areas indicate social interactions and exploratory behaviors. ***C***–***H***, Red traces (±SEM in shaded lines) represent fiber photometry recordings of RCaMP2 signals from OXT^PVN^ neurons represented as *z*-scores in female (***C***, ***D***) and male (***F***, ***G***) mice during social sniffing of familiar and unfamiliar conspecifics. Light orange boxes indicate the baseline period, and dotted vertical lines indicate the time of sniffing initiation by experimental mice (*t* = 0 s). Plots to the right of traces show baseline (BL) and peak responses for each trial (gray lines) and average responses (±SEM) in black dots. A significant Ca^2+^ level change in OXT^PVN^ is observed only in females while interacting with unfamiliar mice. ***E***, ***H***, Plots compare the area under the curve (AUC) and time of peak for Ca^2+^ changes associated with social sniffing of familiar versus unfamiliar conspecifics in males and females. ***I***–***N***, Green traces (±SEM in shaded lines) represent fiber photometry recordings of GCaMP6f signals from astrocytes represented as *z*-scores in female (***I***, ***J***) and male (***L***, ***M***) mice during social sniffing of familiar versus unfamiliar conspecifics in males and females. No change in astrocyte signal was associated with social interactions regardless of sex or conspecific familiarity. ***K***, ***N***, Plots compare AUC and time of peak for Ca^2+^ changes associated with social sniffing of familiar versus unfamiliar conspecifics in males and females. *n* = 5–6 mice, 20–23 traces. *p < 0.05, no other comparison was statistically significant.

Next, we aligned the above behavioral data with Ca^2+^ signals recorded with fiber photometry to analyze Ca^2+^ changes in OXT^PVN^ neurons and astrocytes associated with social investigation. We first examined whether mice exhibited a significant increase in Ca^2+^ levels near the sniff initiation (*t* = 0 s), where peak neuronal activity is observed during social interactions (Solié et al., 2022). We found a significant increase in OXT^PVN^ neuronal Ca^2+^ levels specifically within this time period while female mice interacted with an unfamiliar conspecific (Fig. 3*B*,*D*; Δ*z*-scores at peak, 0.17 ± 0.07; *p* = 0.017, *n* = 19 interactions; paired *t* test), but not with a familiar conspecific (Fig. 3*A*,*C*; Δ*z*-scores at peak, 0.12 ± 0.01; *p* = 0.24, *n* = 20 interactions; paired *t* test). Furthermore, the AUC of Ca^2+^ signal change around sniff initiation was significantly different from zero during unfamiliar (*p* = 0.04, Wilcoxon signed rank test), but not during familiar (*p* = 0.08) interactions. However, when comparing the AUC of the Ca^2+^ signal increase around sniff initiation, we did not find a significant difference between interactions with familiar and unfamiliar mice (Fig. 3*E*; 0.91 ± 0.54, *n* = 20 interactions, and 0.80 ± 0.43, *n* = 19 interactions, respectively; *p* = 0.86, Mann–Whitney test). In accordance with previous findings (Solié et al., 2022), we observed the peak of Ca^2+^ elevations near the onset of sniff initiation (*t* = 0 s) in female mice when interacting with an unfamiliar conspecific. Although there was no statistical difference in the average time of Ca^2+^ peak between the two groups [Δ*t* from sniff initiation in s; 1.6 ± 0.9, *n* = 20 interactions (familiar); 0.16 ± 0.56, *n* = 19 interactions (unfamiliar); *p* = 0.24], the variance in time of peak was lower during interactions with unfamiliar conspecifics compared with that during interactions with familiar conspecifics (*F* = 2.879, Dfn = 19, Dfd = 18; *p* = 0.029, *F* test), indicating a narrower distribution of Ca^2+^ peaks associated with time of sniff initiation for novel social stimuli. These findings could explain the above observation of a lack of significant Ca^2+^ increase within the immediate time window around sniff initiation during interactions with a familiar conspecific.

In male mice, average peak Ca^2+^ changes in OXT^PVN^ neurons during sniff initiation with familiar (Fig. 3*A*,*F*) or unfamiliar (Fig. 3*B*,*G*) conspecifics were not different from one another [Δ*z*-scores at peak, −0.10 ± 0.11, *p* = 0.37, *n* = 20 interactions (familiar); −0.17 ± 0.09, *p* = 0.09, *n* = 21 interactions (unfamiliar); paired *t* test]. Similarly, the median AUCs of Ca^2+^ signal changes associated with interactions with familiar or unfamiliar mice were both in the negative direction [Fig. 3*H*; −9.51 ± 0.82, *n* = 20 interactions (familiar); −14.91 ± 0.85, *n* = 21 interactions (unfamiliar)], although not significantly different from zero (*p* = 0.73 and *p* = 0.10, respectively, Wilcoxon signed rank test). They were not significantly different from each other (*p* = 0.45, Mann–Whitney test). Finally, there was no difference between the time of Ca^2+^ peak between the two groups [1.21 ± 0.71 s (familiar), *n* = 20 interactions; 3.30 ± 1.02 s, *n* = 21 interactions (unfamiliar); *p* = 0.21; Mann–Whitney test]. Together, these results suggest that the dynamic change in Ca^2+^ levels of OXT^PVN^ neurons during social investigation is most prominent in female mice while investigating an unfamiliar conspecific. Moreover, our observation that significant Ca^2+^ changes in OXT^PVN^ neurons occur near the sniffing initiation of unfamiliar interactions in female mice further supports the important role of OXT in encoding novel social information (Oettl et al., 2016).

Finally, we examined whether changes in astrocyte Ca^2+^ levels are associated with social investigation. Unexpectedly, we did not observe any significant changes in astrocyte Ca^2+^ levels in any groups [Fig. 3*A*,*B*,*I*,*J*,*L*,*M*; females, Δ*z*-scores at peak, −0.106 ± 0.069, *p* = 0.1423, *n* = 20 (familiar); 0.046 ± 0.075, *p* = 0.55, *n* = 19 (unfamiliar); males, Δ*z*-scores at peak, −0.149 ± 0.071, *p* = 0.050, *n* = 20 (familiar); −0.244 ± 0.149, *p* = 0.117, *n* = 21 (unfamiliar); paired *t* test]. Interestingly, a slow and steady decrease in signal was observed in male mice when interacting with unfamiliar conspecific mice, although it was not significantly different near sniff initiation (Fig. 3*M*). There were no differences in the AUC or time of peak Ca^2+^ changes associated with conspecific familiarity in either females [Fig. 3*K*; −0.55 ± 0.44, *n* = 20 interactions (familiar); 0.32 ± 0.44, *n* = 19 interactions (unfamiliar); *p* = 0.11, Mann–Whitney test] or males [Fig. 3*N*; −1.11 ± 0.71, *n* = 20 interactions (familiar); −4.11 ± 1.93, *n* = 21 interactions (unfamiliar); *p* = 0.58, Mann–Whitney test]. Similarly, the median time of Ca^2+^ peak was not different between interactions with familiar mice and unfamiliar mice in either females [Fig. 3*K*; Δ*t* from sniff initiation in s; −0.02 ± 0.64, *n* = 20 interactions (familiar); 0.16 ± 0.46, *n* = 19 interactions (unfamiliar); *p* = 0.71, Mann–Whitney test] or males [Fig. 3*N*; Δ*t* from sniff initiation in s; 3.22 ± 1.22, *n* = 20 interactions (familiar); 2.52 ± 1.09, *n* = 21 interactions (unfamiliar); *p* = 0.65, Mann–Whitney test]. Furthermore, the median AUC of peak Ca^2+^ signal was not different from zero in either females [*p* = 0.26 (familiar), *p* = 0.31 (unfamiliar), Wilcoxon signed rank test] or males [*p* = 0.06 (familiar), *p* = 0.45 (unfamiliar), Wilcoxon signed rank test]. These results indicate that social investigation, regardless of sex or familiarity, does not significantly shift Ca^2+^ levels in astrocytes beyond baseline levels.

### Choice of behavioral strategy determines OXT^PVN^ neuronal and astrocyte responses triggered by looming shadow stress

**Figure 4.**
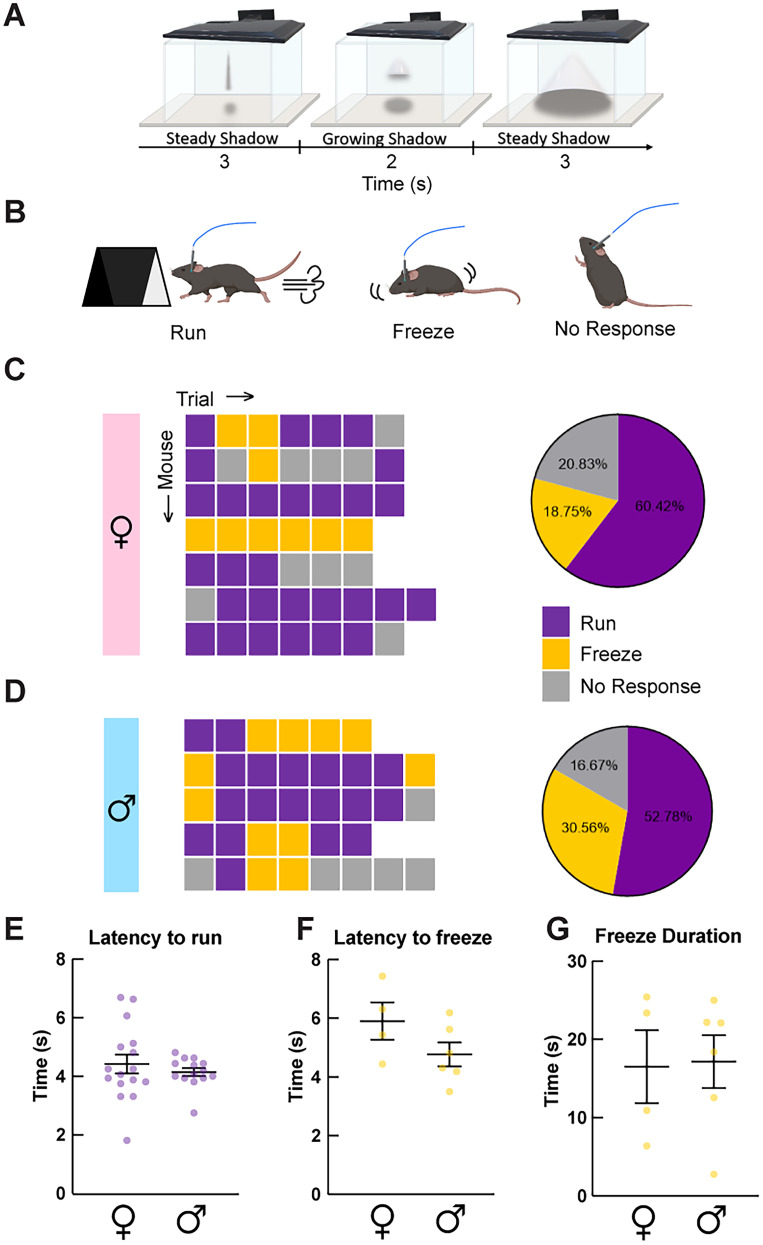
Looming shadow stress task schematics and behavioral results. ***A***, A schematic of the looming shadow stress task paradigm illustrating the phases of shadow presentation from the monitor placed above the experimental arena. ***B***, Mice exhibit three different response categories (i.e., run, freeze, and no response) during the looming shadow task. Created with BioRender.com. ***C***, ***D***, Colored boxes (left) and pie charts (right) represent the behavioral responses displayed by female (***C***) and male (***D***) mice across trials. ***E***, ***F***, Plots (mean ± SEM) with individual trial values compare the latencies and durations of looming shadow-triggered behavioral responses between females and males. No sex differences were found when comparing the latency to run (***E***), latency to freeze (***F***), and freeze duration (***G***).

We next examined Ca^2+^ responses in OXT^PVN^ neurons and astrocytes during innate defensive and survival behaviors in mice by employing the looming shadow task (Fig. 4*A*; Yilmaz and Meister, 2013; Daviu et al., 2020). This experimental setup is designed to replicate an impending threat descending from the sky, involving the expansion of a virtual shadow above the experimental arena. As the perceived threat intensifies, mice exhibit defensive behaviors, either running to shelter or freezing in place (Daviu et al., 2020). First, we examined the behavioral responses of mice to the looming shadow stimuli, categorized into run, freeze, or no response (Fig. 4*B*). As shown in Figure 4, *C* and *D*, no overt sex differences were observed [females, *n* = 29 (run), *n* = 9 (freeze), *n* = 10 (no response); males, *n* = 19 (run), *n* = 11 (freeze), *n* = 6 (no response); *p* = 0.45, chi-square test]. Similar to previously reported results (Daviu et al., 2020), a majority of trials triggered the run response in both males and females (57.78 and 60.42%, respectively; Fig. 4*C*,*D*). Other trials elicited freeze (30.56 and 18.75%, respectively) or no response (16.67 and 20.83%, respectively; Fig. 4*C*,*D*). There were no sex differences in the latency to run or freeze [Fig. 4*E*; latency to run, 4.42 ± 0.32 s, *n* = 16 (females); 4.14 ± 0.14 s, *n* = 14 (males); Fig. 4*F*; latency to freeze, 5.91 ± 0.64 s, *n* = 4 (females); 4.77 ± 0.40 s, *n* = 6 (males); *p* = 0.46 and *p* = 0.15, respectively, unpaired *t* test] or total freeze duration [Fig. 4*G*; 16.53 ± 4.66 s, *n* = 4 (females); 17.17 ± 3.38 s; *n* = 6 (males); *p* = 0.91, unpaired *t* test].

**Figure 5.**
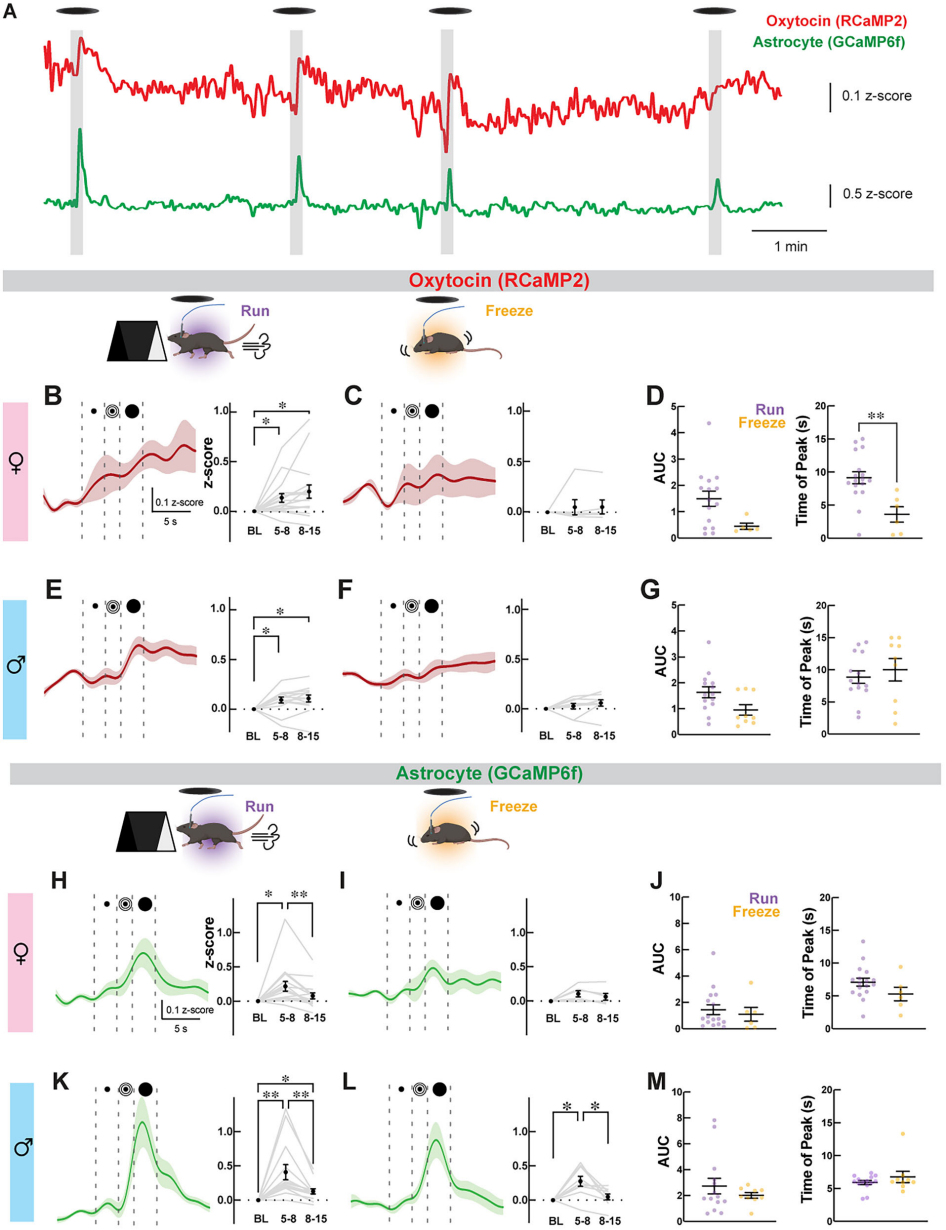
Fiber photometry recordings of OXT^PVN^ neurons and astrocytes during looming shadow task. ***A***, Representative RCaMP2 and GCaMP6f traces from a male exemplar mouse showing Ca^2+^ peaks in response to looming shadow presentations. ***B***–***G***, Traces (±SEM in shaded lines) represent fiber photometry recordings of RCaMP2 signals from OXT^PVN^ neurons represented as *z*-scores in female (***B***, ***C***) and male (***E***, ***F***) mice during looming shadow. Plots to the right of the traces show the average *z*-scores of baseline (BL), looming shadow (5–8 s), and poststimulus (8–15 s) periods for each trial (gray lines) and average responses (±SEM) in black dots. Significant increases in OXT^PVN^ Ca^2+^ levels are observed in both sexes, only during trials with run responses. ***D***, ***G***, Plots compare the area under the curve (AUC) and time of peak for OXT^PVN^ neuron Ca^2+^ changes associated with run and freeze responses in males and females. ***H***–***M***, Traces (±SEM in shaded lines) represent fiber photometry recordings of GCaMP6f signals from astrocytes represented as *z*-scores in female (***H***, ***I***) and male (***K***, ***L***) mice during looming shadow. Significant increases in astrocyte Ca^2+^ level are observed in both run and freeze response trials, except for females during freeze trials. ***J***, ***M***, Plots compare the AUC and time of peak for astrocyte Ca^2+^ changes associated with run and freeze responses in males and females. *p < 0.05, **p < 0.01, all other comparisons were not statistically significant.

A recent study has shown that looming shadow stress stimulus robustly increased the Ca^2+^ level in corticotropin-releasing hormone (CRH) neurons in the PVN, most pronounced during an active defensive behavioral response where mice ran to shelter (Daviu et al., 2020). Given the stress-buffering role of OXT^PVN^ neurons (Windle et al., 1997; Amico et al., 2004, 2008; Mantella et al., 2005; Zoicas et al., 2014), we examined whether the same stress stimulus also increased the Ca^2+^ levels in OXT^PVN^ neurons and whether this response was dependent on the chosen behavioral strategy (i.e., run, freeze, or no response). We found that looming shadow significantly increased OXT^PVN^ neuronal Ca^2+^ levels in trials where mice ran toward the shelter in both females (Fig. 5*B*; Δ*z*-scores; 5–8 s, 0.14 ± 0.044; *Q* = 0.0038, *n* = 15 trials, one-way ANOVA followed by FDR correction) and males (Fig. 5*E*; Δ*z*-scores; 5–8 s, 0.093 ± 0.031; *Q* = 0.0054, *n* = 14 trials). Interestingly, we observed a sustained OXT^PVN^ neuronal Ca^2+^ response in males (Fig. 5*E*; 0.11 ± 0.035; *Q* = 0.13) and even a steady increase in females (Fig. 5*B*; 0.21 ± 0.067; *Q* = 0.032) after the disappearance of the visual stimuli (*t* = 8–15 s). In contrast, trials that triggered the freeze behavior did not affect the Ca^2+^ level of OXT^PVN^ neurons in both females and males (Fig. 5*C*,*F*; Δ*z*-scores; females, 5–8 s, 0.051 ± 0.075; 8–15 s, 0.053 ± 0.069; *Q* = 0.82, *n* = 5 trials; males, 5–8 s, 0.028 ± 0.029; 8–15 s, 0.059 ± 0.032; *Q* = 0.39, *n* = 9 trials).

Next, we further examined whether there is a difference in OXT^PVN^ neuronal Ca^2+^ changes between run and freeze responses to looming shadow. We first compared the average Δ*z*-scores at *t* = 5–8 s between the two behavioral responses within each male mouse and found no statistically significant difference, whether all Δ*z*-scores were averaged for each response [0.075 ± 0.056 (run); 0.044 ± 0.058 (freeze); *p* = 0.53, *n* = 5 mice; two-tailed paired *t* test] or only the first Δ*z*-scores for each behavioral response were compared [0.22 ± 0.068 (run); −0.0036 ± 0.047 (freeze); *p* = 0.49, *n* = 5 mice]. Due to the small number of female mice (*n* = 2) displaying both run and freeze responses, we could not perform the same analysis in this group. Next, we examined the AUCs of Ca^2+^ responses. Significant Ca^2+^ responses were triggered by both run and freeze trials in males [Fig. 5*G*; AUCs, 1.64 ± 0.21, *p* = 0.001, *n* = 14 trials (run); 0.95 ± 0.20, *p* = 0.004, *n* = 9 trials (freeze); Wilcoxon signed rank test), but only in run trials in females [Fig. 5*D*; 1.49 ± 0.29, *p* < 0.001, *n* = 15 (run) trials; 0.26 ± 0.12, *p* = 0.06, *n* = 5 trials (freeze)]. Similar to the Δz-score analysis, no significant differences were detected in the AUC of Ca^2+^ responses between run and freeze trials in either females (Fig. 5*D*, *p* = 0.053; Mann–Whitney test) or males (Fig. 5*G*, *p* = 0.053), but the *p*-values were extremely close to the significance threshold. Moreover, there was a female-specific difference in the time of peak Ca^2+^ response between the run and freeze behavioral responses (Fig. 5*D*; Δ*t* from shadow appearance in s, 9.15 ± 0.90, *n* = 17 trials (run); 3.6 ± 1.16, *n* = 6 trials (freeze); *p* = 0.003; Mann–Whitney test). This difference was absent in males (Fig. 5*G*; 8.87 ± 0.97, *n* = 14 trials (run); 10.01 ± 1.75, *n* = 9 trials; *p* = 0.47; Mann–Whitney test). Finally, no significant Ca^2+^ change in OXT^PVN^ neuron was observed in trials where there was no response to the shadow stimulus (Δ*z*-scores; 5–8 s, 0.015 ± 0.052; 8–15 s, 0.054 ± 0.046; *Q* = 0.82, *n* = 15, both sexes combined). These results suggest that strong and persistent increases in OXT^PVN^ neuronal activity are generally associated with an active escape strategy (i.e., run responses) during the looming shadow stress in both sexes.

We detected a robust increase in astrocyte Ca^2+^ levels during the looming shadow trials. The stimulus response peaked during the last phase of looming shadow presentation (*t* = 5–8 s), remarkably similar to the reported response characteristics of CRH neurons under the same stimulus (Daviu et al., 2020). Specifically, run responses were associated with significant Ca^2+^ increases in both male and female mice when we compared *z*-scores between the baseline and looming shadow periods [Fig. 5*H*,*I*; Δ*z*-scores; 5–8 s, 0.41 ± 0.11; 8–15 s, 0.13 ± 0.037 (males); 5–8 s, 0.22 ± 0.071; 8–15 s, 0.077 ± 0.043 (females); *Q* < 0.0001, one-way ANOVA followed by FDR correction]. In contrast, freeze response trials were associated with significantly increased astrocyte Ca^2+^ levels in male mice (Fig. 5*L*; Δ*z*-scores; 5–8 s, 0.28 ± 0.073; 8–15 s, 0.48 ± 0.044; *Q* = 0.0052; *n* = 9), but not in female mice (Fig. 5*I*; Δ*z*-scores; 5–8 s, 0.11 ± 0.045; 8–15 s, 0.065 ± 0.049; *Q* = 0.12; *n* = 6). Directly comparing astrocyte Ca^2+^ changes between run and freeze responses to looming shadow, no differences in average Δ*z*-scores (*t* = 5–8 s) were found between the two behavioral responses within each male mouse, whether all Δ*z*-scores were averaged for each response [0.47 ± 0.21 (run); 0.21 ± 0.073 (freeze); *p* = 0.15, *n* = 5 mice; two-tailed paired *t* test] or only the first Δ*z*-scores for each behavioral response were compared [0.60 ± 0.28 (run); 0.19 ± 0.11 (freeze); *p* = 0.11, *n* = 5 mice]. AUC analysis of stimulus-triggered Ca^2+^ changes revealed significant increases in both male [AUC: *p* = 0.0001 (run), *p* = 0.004 (freeze); Wilcoxon signed rank test] and female [AUC: *p* < 0.0001 (run), *p* = 0.03 (freeze)] mice regardless of behavioral responses. In agreement with the peak *z*-score comparisons, no significant differences were detected in the AUC or the time of peak of Ca^2+^ responses between run and freeze trials in either females [Fig. 5*J*; AUC, 1.45 ± 0.38, *n* = 16 (run); 1.10 ± 0.53, *n* = 6 (freeze); *p* = 0.54; Mann–Whitney test; time of peak, 7.07 ± 0.65 (run); 5.30 ± 1.04 (freeze); same *n* values as AUC, *p* = 0.12] or males [Fig. 5*M*; AUC, 2.73 ± 0.61, *n* = 14 (run); 2.02 ± 0.22, *n* = 9 (freeze); *p* > 0.99; Mann–Whitney test; time of peak, 5.92 ± 0.30 (run); 6.76 ± 0.84 (freeze); same *n* values as AUC, *p* = 0.93], which suggests the existence of a low magnitude Ca^2+^ response in female mice displaying freeze responses that could not be detected using our peak analysis method. Unlike the Ca^2+^ response in OXT^PVN^ neurons, the disappearance of shadow stimulus was associated with a sharp decline in astrocyte Ca^2+^ levels back to baseline in both run and freeze responses (Fig. 5*H*,*K*,*L*). Finally, no significant change in astrocyte Ca^2+^ levels was observed in trials where a behavioral response to the shadow stimulus was absent (not shown; Δ*z*-scores; 5–8 s, 0.014 ± 0.03; 8–15 s, −0.047 ± 0.037; *Q* = 0.67), confirming that astrocyte Ca^2+^ responses are associated with the presence of a defensive behavioral response (i.e., run or freeze) to the stress stimulus. In summary, the Ca^2+^ responses observed in astrocytes greatly resemble those reported for CRH neurons (Daviu et al., 2020), with the maximum peak occurring during the last stage of the looming shadow stimulus (*t* = 5–8 s), followed by a return to baseline poststimulus. In contrast, this response pattern was not observed in OXT^PVN^ neurons, which instead exhibit a sustained Ca^2+^ elevation throughout the last stage of the looming shadow stimulus and the poststimulus (*t* = 8.01–15 s) period (Fig. 5*B*,*E*).

### The functional coupling between OXT^PVN^ neurons and astrocytes is sex- and context-dependent

**Figure 6.**
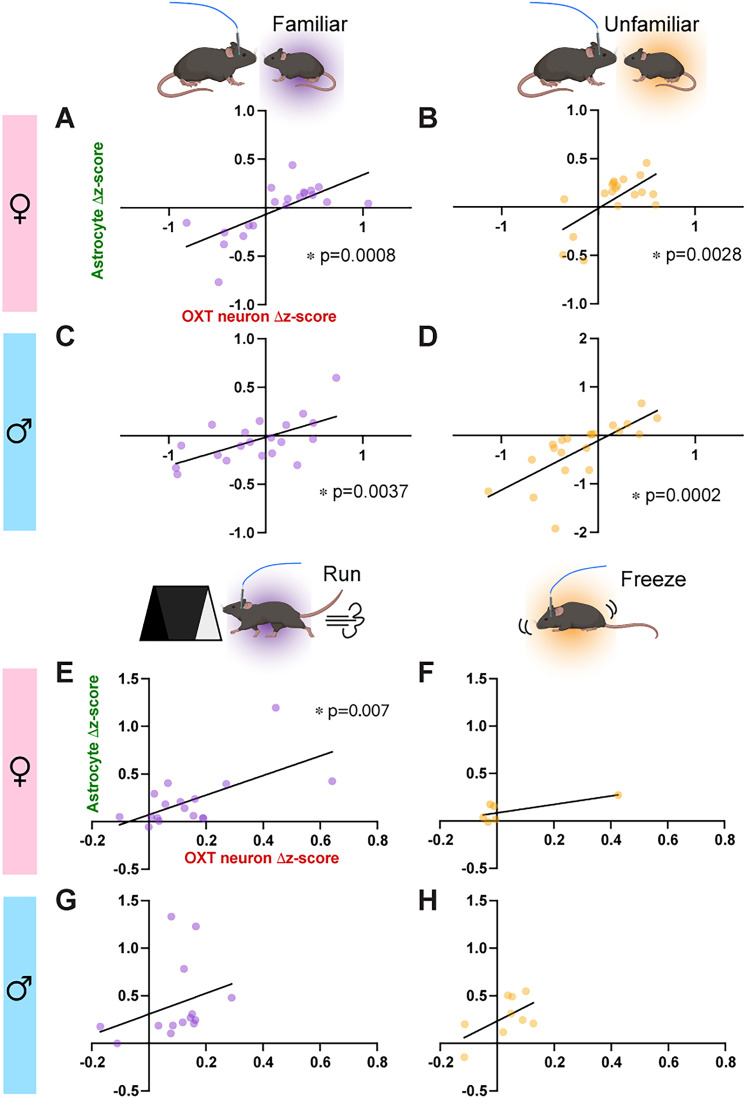
Correlated Ca^2+^ changes between OXT^PVN^ neurons and astrocytes during home cage social interactions and looming shadow stress. Scatter plots and linear regression lines compare the correlational relationship in stimulus-associated Ca^2+^ changes between astrocyte and OXT^PVN^ neurons in male and female mice during social interactions (***A***–***D***) and looming shadow stress (***E***–***G***). *p < 0.05, all other linear regression showed no significant association between astrocyte and OXT^PVN^ neurons.

Finally, we investigated the correlation between OXT^PVN^ neurons and astrocyte Ca^2+^ changes to assess whether functional coupling exists between the two cell types during home cage social interaction and looming shadow stress task. To examine this, we identified the time at which stimulus-associated Ca^2+^ change in OXT^PVN^ neurons peaked for each trial and compared the magnitude of Ca^2+^ changes between astrocytes and OXT^PVN^ neurons. A simple linear regression analysis showed significant positive correlative relationships between the magnitude of Ca^2+^ peaks in OXT^PVN^ neurons and astrocytes in female mice while interacting with familiar (Fig. 6*A*; *R*^2^ = 0.48, *y* = 0.41x − 0.068, *p* = 0.0008) and unfamiliar (Fig. 6*B*; *R*^2^ = 0.42, *y* = 0.60x − 0.014, *p* = 0.0028) conspecifics. Likewise, significant positive correlations were observed in males during interaction with both familiar (Fig. 6*C*; *R*^2^ = 0.38, *y* = 0.29x − 0.015, *p* = 0.0037), and unfamiliar (Fig. 6*D*; *R*^2^ = 0.53, *y* = 1.0x − 0.11, *p* = 0.002) conspecifics. During the looming shadow task, a significant positive correlation was exclusively observed when female mice exhibited a run response toward the shelter (Fig. 6*E*; *R*^2^ = 0.40, *y* = 1.03x + 0.072, *p* = 0.0069). No significant correlations in Ca^2+^ changes were observed in all other groups (Fig. 6*F*–*H*; female: freeze, *R*^2^ = 0.55, *y* = 0.45x + 0.084, *p* = 0.091; males: run, *R*^2^ = 0.095, *y* = 1.095x + 0.31, *p* = 0.28, freeze: *R*^2^ = 0.36, *y* = 1.51x + 0.23, *p* = 0.095). These results suggest sex- and context-specific interplay between OXT^PVN^ neurons and astrocytes.

## Discussion

Using dual-color fiber photometry in freely behaving mice, here we report sex- and context-dependent response patterns of OXT^PVN^ neurons and neighboring astrocytes in social and stress contexts. In the social context, female interactions with unfamiliar conspecifics were associated with a significant increase in Ca^2+^ levels of OXT^PVN^ neurons near sniff initiation without any detectable Ca^2+^ change in astrocyte (Fig. 3). In contrast, male interactions did not significantly change the Ca^2+^ levels of either OXT^PVN^ neurons or astrocytes (Fig. 3). In the stress context, looming shadow-triggered run responses in male and female mice were associated with increased activity in both OXT^PVN^ neurons and astrocytes, although the response dynamics of the two cell types were distinct (Fig. 5). In contrast, only males exhibited prominent Ca^2+^ changes associated with freeze responses (Fig. 5). Furthermore, OXT^PVN^ neurons and astrocytes exhibited significantly correlated stimulus-associated Ca^2+^ changes during social investigations of conspecifics in all groups (Fig. 6). On the other hand, only run responses by female mice under the looming shadow stress were associated with significantly correlated Ca^2+^ changes between OXT^PVN^ neurons and astrocytes (Fig. 6). These findings suggest that OXT^PVN^ neurons and astrocyte activity, as well as their functional coupling, are highly selective and sex-dependent under social and stress contexts, providing a greater understanding of the dynamic activity relationship between the two cell types that is linked to behavioral outcomes.

A previous two-photon imaging study has demonstrated that social stimuli can increase or decrease the activity of OXT^PVN^ neuronal subsets (Resendez et al., 2020), which perhaps supports observations from a recent fiber photometry study where social stimuli only mildly increased OXT^PVN^ neuronal activity at the population level (Zhan et al., 2024). Consistent with these results, we observed a modest and short-duration (∼2 s long with a peak near sniffing onset) increase in OXT^PVN^ neuronal Ca^2+^ levels time-locked to sniffing initiation in females while interacting with unfamiliar conspecifics (Fig. 3*D*,*E*). Although social sniffing familiar conspecifics also appeared to increase Ca^2+^ in female mice, the time of peak was significantly more variable compared with that during interactions with an unfamiliar conspecific (Fig. 3*E*). In male mice, we did not detect consistent Ca^2+^ changes in OXT^PVN^ neurons near sniffing initiation regardless of the familiarity of the conspecifics (Fig. 3*F*,*G*). These conspecific familiarity-dependent activity changes we demonstrate in both sexes are consistent with the known role of OXT^PVN^ neuronal activity in encoding of social information (Oettl et al., 2016), which contributes to social recognition (Ferguson et al., 2001; Gabor et al., 2012; Oettl et al., 2016). Furthermore, these results identify the potential existence of a female-specific mechanism used by OXT^PVN^ neurons that enables a coordinated response that is time-locked to new social encounters. Although the small magnitude of Ca^2+^ changes associated with social sniffing is not unique to our study, the use of RCaMP2, which has a lower maximum Δ*F*/*F* compared with GCaMPs including GCaMP6f (Inoue et al., 2015) may have prevented visualization of potentially important Ca^2+^ fluctuations in OXT^PVN^ neurons during social interactions. Future development of even more sensitive Ca^2+^ sensors may reveal novel population activity patterns of OXT^PVN^ neurons that may be physiologically relevant. Finally, since our method relied on Oxt-Cre–dependent expression of RCaMP2 to detect changes in Ca^2+^ levels, it is not possible to attribute our results to either magnocellular or parvocellular population of OXT^PVN^ neurons. However, we targeted the parvocellular-rich midcaudal region of the PVN to ensure that Ca^2+^ signals from parvocellular neurons are included in our study due to their important role in social processes including social reward learning (Lewis et al., 2020). Whether parvocellular OXT^PVN^ neurons indeed specifically contribute to encoding the familiarity of conspecifics, and therefore social discrimination, would be an interesting avenue for future investigations.

Looming shadow stress triggered a stronger and longer-lasting increase in OXT^PVN^ neuronal Ca^2+^ levels (persisting even >7 s after looming shadow disappearance) in both sexes (Fig. 5). These results are also consistent with the above fiber photometry study that reported stronger activation of OXT^PVN^ neurons by stressors (Zhan et al., 2024). It is important to note that this previous study used high-intensity stressors that also provide strong touch sensations, such as footshocks, air puffs, tail suspension, restraint, and social aggression. Given that OXT^PVN^ neurons are robustly activated by touch (Tang et al., 2020), it is possible that the touch component of the stressors used in the study may have significantly contributed to the robust responses that the authors observed in OXT^PVN^ neurons. Our study, for the first time, demonstrates that touch is not necessary to elicit stress-induced increases in OXT^PVN^ neuronal Ca^2+^ levels using a visual stress stimulus resembling an aerial predator threat. Furthermore, we demonstrate that the Ca^2+^ changes in OXT^PVN^ neurons are specific to the behavioral response strategy chosen by the mouse—significant increases were observed in trials with run responses but absent in those with freeze responses (Fig. 5). The superior colliculus has been identified as a critical structure for integrating inputs into hypothalamic circuits, with direct projections to the PVN (Carcea et al., 2021; Son et al., 2022; Menon and Neumann, 2023). These projections suggest a potential mechanism by which the looming shadow stimulus might activate OXT^PVN^ neurons. The direct pathway, mediated by superior colliculus–PVN projections (Carcea et al., 2021), could allow for the rapid integration of visual information to the PVN, enabling immediate behavioral response to a perceived threat, such as the run response. Alternatively, the indirect pathway, which could involve the thalamus and amygdala (Wei et al., 2015), could modulate OXT^PVN^ neuronal activity during the freeze response.

The long-lasting nature of OXT^PVN^ neuronal Ca^2+^ level increases under the looming shadow stimulus is intriguing and contrasts with the response profile of PVN CRH neurons that sharply declines after the disappearance of the shadow stimulus (Daviu et al., 2020). Future studies may be able to identify the cellular mechanisms underlying these differences in Ca^2+^ dynamics and investigate how the sustained Ca^2+^ elevation in OXT^PVN^ neurons may promote effective stress buffering. In an in vitro experiment, OXT was shown to suppress excitatory synaptic input to CRH neurons (Jamieson et al., 2017), suggesting that stress-induced Ca^2+^ elevation in OXT^PVN^ neurons that we report here (Fig. 5*B*,*E*) could lead to an inhibition of CRH neurons through dendritically released OXT. If this is the case, experimentally blocking the dendritic release of OXT, such as through inhibiting Ca^2+^ elevations (Ros et al., 2020) or exocytosis through upregulating synaptotagmin-4 (Zhang et al., 2011) in OXT^PVN^ neurons, would further increase CRH neuronal activity during looming shadow stress. Furthermore, considering the sustained nature of Ca^2+^ elevation in OXT^PVN^ neurons (Fig. 5*B*,*E*), blocking this transmission could delay the decline of CRH activity to baseline poststimulus. This could reveal a potential stress-buffering mechanism by which OXT^PVN^ neurons locally regulate CRH neuronal activity.

In contrast to OXT^PVN^ neurons that were activated by both social and stress stimuli, PVN astrocytes were only activated by stress stimuli (Fig. 5) and not social stimuli (Fig. 3). These results suggest that PVN astrocytes may be more sensitive to stress stimuli compared with social stimuli. Indeed, the looming shadow stimulus robustly increased astrocyte Ca^2+^ levels in all groups except for trials with freeze responses in females (Fig. 5*H*–*M*). The comparatively stronger activation of PVN astrocytes during the looming shadow stimulus could be representative of astrocytes’ ability to report global, coordinated changes in neuronal activity in multiple PVN neuronal cell types, including CRH and OXT neurons. Indeed, the Ca^2+^ dynamic of astrocytes, across all conditions where significant responses were observed, was strikingly similar to previously reported results describing CRH neuron responses to looming shadow stress stimulus delivered identically to our study (Daviu et al., 2020). This is perhaps not surprising, given the previous in vitro finding that norepinephrine activation of CRH neurons can trigger astrocyte Ca^2+^ responses via vasopressin as a messenger between the two cell types (Chen et al., 2019). While our study serves as the first in vivo demonstration of this potential cross talk between CRH neurons and astrocytes during stress, another study has shown similar Ca^2+^ changes in hippocampal astrocytes during footshock stimulations (Suthard et al., 2024). Given this potential CRH neuron–astrocyte cross talk during stress, we speculate that the robust Ca^2+^ response in astrocytes we report here could be the result of simultaneous activation of OXT and CRH neurons in response to the stress stimuli. Due to the correlative nature of this study, it is impossible to discern whether the astrocyte response to looming shadow stress we report here contributes to the observed OXT^PVN^ neuronal response. Future in vitro and in vivo studies using optogenetics and pharmacology will reveal greater insights into whether a cross talk between OXT^PVN^ neurons and astrocytes actually exists under a heightened level of norepinephrine (e.g., during stress), which may contribute to the mechanism underlying stress-mediated release of OXT and its stress-buffering effects.

Our observation of significant correlations in stimulus-associated Ca^2+^ level change between OXT^PVN^ neurons and astrocytes is well supported by the extremely close structural and functional relationship that astrocytes have with hypothalamic neurons (Panatier et al., 2006; Baudon et al., 2022), including OXT neurons (Wang and Hatton, 2009). In both familiar and unfamiliar interactions, we observed significant correlations between OXT^PVN^ and astrocyte Ca^2+^ changes in females and males (Fig. 6). These activity correlations suggest functional coupling between the two cell types, potentially mediated by somatodendritic release of OXT under stimulated conditions (Ludwig, 1998; Ludwig and Leng, 2006; Grinevich and Neumann, 2021). In this context, activation of OXT receptors expressed on neighboring astrocytes would lead to increased intracellular Ca^2+^ levels in these cells via activation of downstream G-protein signaling pathways (Baudon et al., 2022; Meinung et al., 2024), a mechanism recently demonstrated to mediate the anxiolytic effects of OXT in the PVN (Meinung et al., 2024). Interestingly, activation of the OXT receptor has been shown to trigger astrocyte structural plasticity (Baudon et al., 2024; Meinung et al., 2024). In particular, stress has been shown to induce an OXT receptor–Gαi pathway-dependent retraction of astrocyte processes in the amygdala, potentially indicating a structural plasticity mechanism that reduces the degree of neuron–astrocyte functional coupling (Baudon et al., 2024). Indeed, our results indicate that Ca^2+^ changes in OXT^PVN^ neurons and neighboring astrocytes under looming shadow stress became uncoupled in male mice, regardless of the behavioral response (Fig. 6*G*,*H*). In support of these findings, a recent fiber photometry study has also reported significant activity correlations between hippocampal neurons and astrocytes, whose coupling strengths vary depending on learned fear contexts (Suthard et al., 2024). Whether these forms of astrocyte–neuron functional decoupling involve similar structural plasticity in astrocytes remains to be explored.

The potential functional decoupling between OXT^PVN^ and neighboring astrocytes under the looming shadow stimulus underscores their differences in Ca^2+^ dynamics in response to stress induced by perceived predatory threat. While astrocytes could be closely tracking the activity of OXT^PVN^ neurons during social interactions, their Ca^2+^ response during looming shadow may be more strongly influenced by the activity of CRH neurons also located within the PVN and robustly stimulated by looming shadow (Daviu et al., 2020). It would be interesting to examine the impact of social stress, such as repeated social defeat (Golden et al., 2011), on the functional coupling between OXT^PVN^ neurons and neighboring astrocytes. In this scenario, astrocytes may respond to the stress stimuli while OXT^PVN^ neurons primarily respond to the social stimuli. This separate response could prevent astrocytes from being further stimulated by OXT^PVN^ neurons due to the already heightened level of Ca^2+^. Previous studies have demonstrated that stress associated with social interactions can activate the HPA axis—in humans, cortisol levels are higher during interactions with strangers compared with partners (Handlin et al., 2023). In mice, social interactions with unfamiliar conspecifics elevate the activity of hypocretin neurons in the lateral hypothalamus (Dawson et al., 2023), subsequently exciting CRH neurons (Bonnavion et al., 2015). Therefore, elevated levels of stress may create a decoupling of the functional relationship between the two cell types and potentially have downstream impacts on physiology and behavior.

A major finding of our study is the robust sex differences in the Ca^2+^ changes in OXT^PVN^ and astrocytes observed across different social and stress contexts. Clear sex differences in OXT expression have been reported across various rodent species (Dumais and Veenema, 2016). Furthermore, various sex hormones and their metabolites, such as estrogen (Akaishi and Sakuma, 1985), testosterone (Israel et al., 2014), and allopregnanolone (Brussaard et al., 1997), regulate the activity of OXT neurons. We did not observe overt sex differences in OXT^PVN^ neuronal response to looming shadow stress (Fig. 5*B*–*G*). However, the downstream brain regional effects of stress-triggered OXT^PVN^ neuronal activation may still vary by sex. For example, the modification of estrogen receptor beta function by female reproductive hormones in the PVN promotes the production of OXT mRNA, leading to additional decreases in anxiety-like behavior and dampening of stressed-triggered corticosterone responses (Nomura et al., 2002; Acevedo-Rodriguez et al., 2015). This mechanism may allow increased central OXT release in females despite similar levels of stress-induced increases in OXT^PVN^ neuronal activity. Given this possibility, it can be postulated that the stage of the estrous cycle may have a significant impact on OXT^PVN^ neuronal activity in female mice under different stress and social contexts. Although we did not assess this factor in our study, it would be important to investigate the influence of the estrous cycle in future studies. Additionally, sex-specific mechanisms for OXT signaling in downstream brain regions may also play a role. For example, OXT signaling in the medial amygdala has been shown to support discrimination of social cues in a sex-specific manner (Yao et al., 2017). Although this observation is restricted to the social domain, the mechanism may also extend to stress-triggered OXT signaling in the amygdala (Knobloch et al., 2012) due to its well-known role in stress and anxiety-like behavior. The estrous cycle is also likely to affect astrocyte interaction with OXT neurons. Astrocytes express estrogen receptors (Azcoitia et al., 1999; Chaban et al., 2004; Pawlak et al., 2005) and sex-specific gene expression patterns (Wilhelm et al., 2016; Rurak et al., 2022). Furthermore, the morphology of hippocampal astrocytes is impacted by the estrous cycle (Arias et al., 2009). Given that astrocyte structure is critical for the functional regulation of neuronal activity, this could impact their functional coupling. Finally, the estrous cycle has been shown to affect social approach (Chari et al., 2020) and anxiety-like behavior (Pestana and Graham, 2024) in female rodents. Whether estrous cycle regulation of OXT^PVN^ neuronal activity and OXT release constitutes a significant component of these behavioral differences would be an important future research direction.

The observed Ca^2+^ changes in OXT^PVN^ neurons associated with social and stress stimuli are expected to cause peripheral and central OXT release, leading to physiological and behavioral effects that are crucial to both physiological regulation and behavioral adaptation. Peripheral OXT has been associated with a reduction in anxiety, as evidenced by studies showing that rats administered peripheral OXT exhibit decreased background anxiety-like behavior (Ayers et al., 2011). In humans, peripheral OXT enhances both sympathetic and parasympathetic cardiac control (Norman et al., 2011), suggesting its utility in stress-related contexts that require adaptive behavioral responses such as freezing or fleeing, as well as during stress recovery phases. Although the effects of peripheral OXT on social interaction remain less well understood, its physiological and behavioral effects associated with stress suggest a potential peripheral component in alleviating anxiety triggered by novel social encounters. Central OXT release has clearly demonstrated anxiolytic effects through the amygdala (Knobloch et al., 2012) as well as reductions in blood pressure (Petersson et al., 1996), which may be particularly relevant for regulating stress responses to threatening stimuli such as the looming shadow stress stimulus. In social contexts, central OXT is well established to enhance the salience of social stimuli during interactions (Johnson and Young, 2017), potentially through mechanisms involving OXT release in brain regions such as the auditory cortex (Marlin et al., 2015), nucleus accumbens (Dölen et al., 2013), hippocampus (Tirko et al., 2018), and olfactory cortex (Oettl et al., 2016).

In summary, our findings highlight the sex- and context-dependent activity of OXT^PVN^ neurons and astrocytes in vivo. Furthermore, these results serve as the first demonstrations of their correlated Ca^2+^ dynamics at the population level in vivo, shedding light on how these two cell types may work together to generate proper physiological and behavioral responses to social or stress stimuli, to ultimately increase the likelihood of survival. These findings also reveal novel insights into the neurobiology of social behavior and stress responses, potentially informing therapeutic strategies targeting conditions associated with disruptions in these processes.

## Synthesis

Reviewing Editor: Jibran Khokhar, Western University Department of Anatomy and Cell Biology Decisions are customarily a result of the Reviewing Editor and the peer reviewers coming together and discussing their recommendations until a consensus is reached. When revisions are invited, a fact-based synthesis statement explaining their decision and outlining what is needed to prepare a revision will be listed below. The following reviewer(s) agreed to reveal their identity: Silvana Valtcheva, Karl Iremonger.

This study uses in vivo dual-color fiber photometry to monitor the activity of PVN oxytocin neurons and astrocytes, finding that oxytocin neurons in female mice are activated by an unfamiliar mouse but not a familiar one, with no changes in male mice. Both sexes show increased oxytocin neuron activity and astrocyte Ca2+ during escape from a looming threat, with astrocytic Ca2+ also significantly rising during freezing in males. Both reviewers were excited about the advances to the field and the relevance to eNeuro readership.

Reviewer 1

Intro and abstract:

Make sure the appropriate claims are made based on the citations used. Several papers NOT concerning oxytocin neurons are cited as studies investigating PVN-OXT cells. Examples: Wang and Hatton, 2009 and Panatiet et al., 2006 made recordings from unidentified magnocellular neurons in SON slices of rats - these cells can be either OXT or vasopressin neurons. Chen et al., 2019 did not record from oxytocin cells but from fluorescently identified PVN-CRH neurons.

Change claims everywhere in the text and write "magnocellular SON/hypothalamic neurons" instead of OXT-PVN neurons. For example line 9 of the abstract: "Astrocytes, a type of glial cells, can bidirectionally interact with OXT-PVN neurons to participate in local activity regulation within the PVN" should be changed to "Astrocytes [...] interact with hypothalamic magnocellular neurons".

Cite appropriate recent studies in vivo/in slice instead of/in addition to studies in cell cultures. Cornell-Bell et al., 1990 for example is a study done in cell culture.

Add in the text that results from Di Scala-Guenot et al., 1994 were obtained from cell culture.

Give literature background addressing astrocyte detection of peptides/neuromodulators, not just glutamate and K+. This is more appropriate when making the link to oxytocin.

Figures:

1) Fig. 1 - add a histology verification of fiber implantation; the graphical representation in Extended Fig. 1-1. is not enough.

2) Fig. 2 - add analysis of area under the curve for photometry data. Oxytocin neurons in the PVN exhibit long-lasting (several seconds to minutes) activity in vivo, demonstrated both by calcium data (Resendez et al., 2020; Rajamani et al., 2021; Yukinaga et al., 2022; Valtcheva et al., 2023; Zhan et al., 2024) and electrophysiology recordings (Tang et al., 2020; Carcea et al., 2021). This suggests that PVN-OXT cells integrate activity over a long timescale and thus, analysis of the area under the curve will give an additional information about neural activity over time.

Discussion:

1) Comment on visual projections to PVN-OXT neurons - superior colliculus projections to PVN in mice (Carcea et al., 2021; Freda et al., 2022 biorxiv; Son et al., 2022; reviewed in Menon &Neumann, 2023).

2) Comment on how direct sensory (visual) vs other mechanisms can contribute to the activation of PVN-OXT neurons during looming shadow.

3) Comment on what are the potential consequences for central vs peripheral oxytocin release.

4) Comment on how estrous cycle may influence behavior results and neural/astrocytic activity.

Reviewer 2

This manuscript uses in vivo dual-color fiber photometry to record the activity of PVN oxytocin neurons and astrocytes. The authors report that oxytocin neurons in female mice show increased activity in response to an unfamiliar mouse, however, no change in response to a familiar mouse. No change in activity was observed in male mice or in astrocyte Ca2+. In response to a looming threat stimulus, the authors report that both female and male mice show increases in oxytocin neuron activity, but only in trials where animals escape from the stimulus. Astrocyte Ca2+ was also found to be increased in response to the escape in males and females but was found only to be significantly changed in response to freezing in males.

This is an interesting and important topic of study. The techniques used are also cutting edge. However, I have several concerns which are detailed below.

1) The two color fiber photometry is a great technique, however, critically depends on the specificity of the viral expression. As such, a more detailed characterisation of the expression of GCaMP and RCaMP is required. One representative image is insufficient.

Co-staining for both GFAP (or another astrocyte marker), GCaMP and RCaMP is required in one set of sections and in another set of sections staining is required for oxytocin, RCaMP and GCaMP. A quantification of colocalization of these markers in stained cells then needs to be performed. Importantly, this should be done in several mice. I think this is critical for the paper.

2) The analysis and presentation of the photometry data could also be improved. Firstly, please show example traces from single mouse recordings at both baseline (no stimulus) and in response to the tested stimulus. These are needed to see the quality of the photometry recordings. Good photometry recordings would reveal distinct Ca2+ events in oxytocin neurons under baseline conditions.

3) For all mean photometry traces, I would like to see longer recordings (especially longer pre-stimulus baselines). The way the analysis is performed for the photometry is also problematic. A single timepoint for the baseline is normalised for all recordings giving values with zero variance and then this is compared to a peak value. This approach really should be avoided.

4) For the loom data, analysis is done on a per trial basis and separated into trials where there was an escape vs freeze or no response. Because there are more escape responses than freeze responses, the authors are biasing their analysis to be able to detect smaller statistical changes in the escape group by virtue of having more data points to analyse (there are more escapes than freezes). To avoid this, analysis should be performed per animal rather than per trial.

5) In the abstract and several other places in the manuscript, it is stated that "Ca2+ levels of OXTPVN neurons and astrocytes were significantly correlated". This gives the impression that they are correlated over time, when in fact the corelation analysis was simply at a single time point and was in terms of the magnitude of the signal. Please reword to more accurately reflect what was found.

6) There were a few methodological details that were missing.

a) Please state if mice were individually or group housed after surgery. How might this have changed physiology/results?

b) Were the excitation light source frequency modulated? If so, what frequencies were used?

c) Please report the details of the excitation and emission filters and dichroic mirrors used.

d) Why were mice habituated in the dark? How long did they stay in the dark? Were any behavioural experiments performed in the dark?

e) How many mice were excluded due to the criteria in the social interaction test? Please report in the manuscript.

7) A couple points for consideration in the discussion section. Could the lack of significant results in the social interaction test be partly caused by a low sensitivity of RCaMP? Could the fact that significant Ca2+ responses were only observed in astrocytes in the loom test be because Ca2+ signals in astrocytes are reporting global neural activity?

8) Figure 5 has an incorrect title.

9) This paper is missing from the reference list (Resendez et al 2020).
