# Supplementary figures and images for: Calcium Dynamics in Hypothalamic Paraventricular Oxytocin Neurons and Astrocytes Associated with Social and Stress Stimuli
Source: eNeuro. 2025 May 8;12(5):ENEURO.0196-24.2025. doi: 10.1523/ENEURO.0196-24.2025 (PMC12071343; doi:10.1523/ENEURO.0196-24.2025)

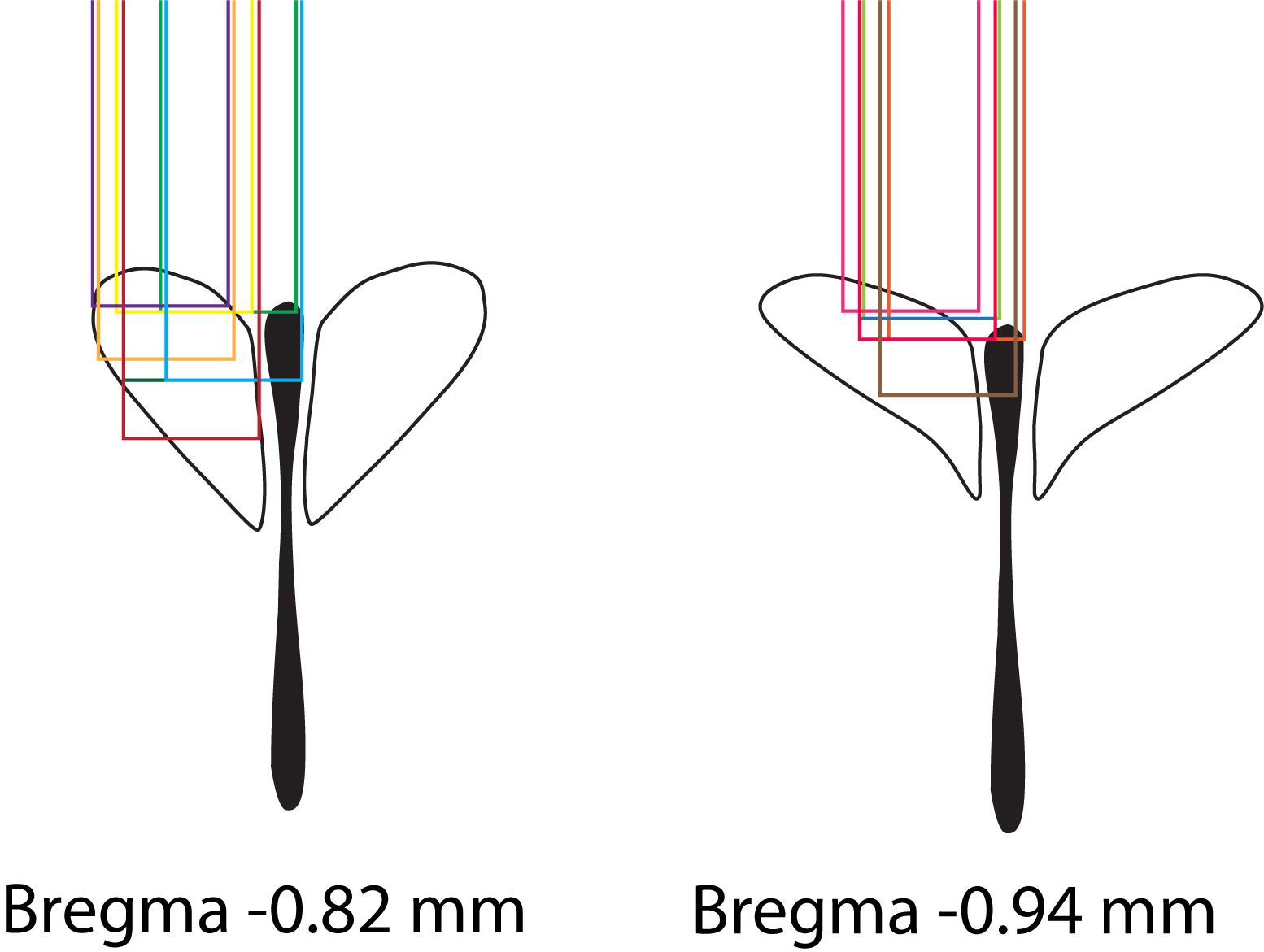

Supplement: Figure 1-1 — Graphical representation of optic fiber implants targeting the PVN. Each color represents an individual mouse. Supports Fig .1. Download Figure 1-1, TIF file. [file eneuro-12-ENEURO.0196-24.2025-s001.tif]

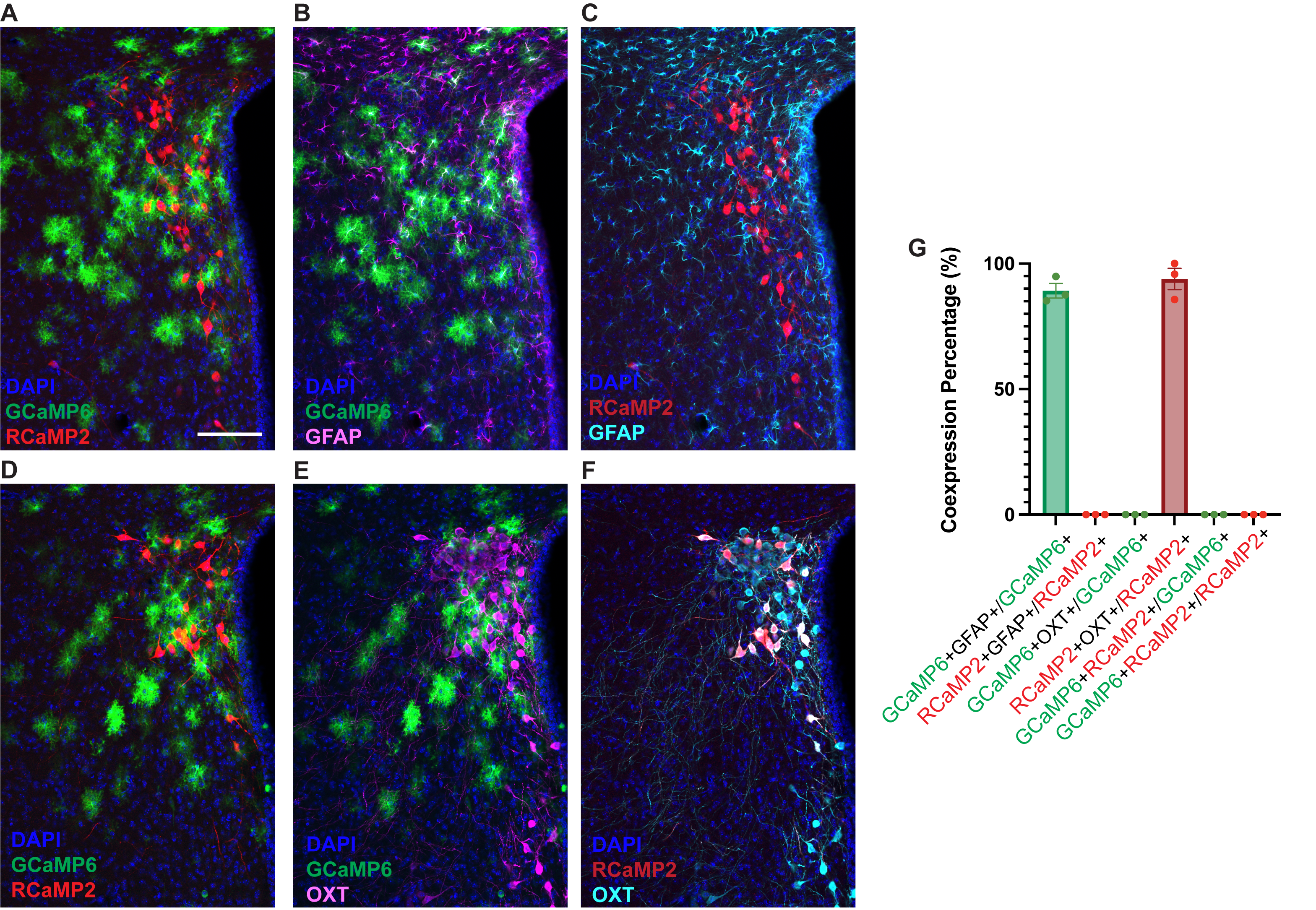

Supplement: Figure 1-2 — Histological confirmation of co-expression specificity for GCaMP6 and RCaMP2. Confocal images of immunostained PVN sections displaying specificity of GCaMP6 expression in GFAP + astrocytes (A-C) and RCaMP2 in OXT-expressing cells (D-F). G, Plot shows a high (∼90%) level of coexpression percentage only for cells expressing both GCaMP6 and GFAP and those expressing RCaMP2 and OXT, but virtually none expressing other combinations. Scale bar = 100 μm. Supports Fig .1. Download Figure 1-2, TIF file. [file eneuro-12-ENEURO.0196-24.2025-s002.tif]
